# Structural basis for proapoptotic activation of Bak by the noncanonical BH3-only protein Pxt1

**DOI:** 10.1371/journal.pbio.3002156

**Published:** 2023-06-14

**Authors:** Dahwan Lim, So-Hui Choe, Sein Jin, Seulgi Lee, Younjin Kim, Ho-Chul Shin, Joon Sig Choi, Doo-Byoung Oh, Seung Jun Kim, Jinho Seo, Bonsu Ku

**Affiliations:** 1 Disease Target Structure Research Center, Korea Research Institute of Bioscience and Biotechnology, Daejeon, Korea; 2 Critical Diseases Diagnostics Convergence Research Center, Korea Research Institute of Bioscience and Biotechnology, Daejeon, Korea; 3 Department of Biochemistry, Chungnam National University, Daejeon, Korea; 4 Aging Convergence Research Center, Korea Research Institute of Bioscience and Biotechnology, Daejeon, Korea; 5 Department of Biosystems and Bioengineering, KRIBB School of Biotechnology, University of Science and Technology, Daejeon, Korea; 6 Department of Proteome Structural Biology, KRIBB School of Bioscience, University of Science and Technology, Daejeon, Korea; Universitat zu Koln, GERMANY

## Abstract

Bak is a critical executor of apoptosis belonging to the Bcl-2 protein family. Bak contains a hydrophobic groove where the BH3 domain of proapoptotic Bcl-2 family members can be accommodated, which initiates its activation. Once activated, Bak undergoes a conformational change to oligomerize, which leads to mitochondrial destabilization and the release of cytochrome *c* into the cytosol and eventual apoptotic cell death. In this study, we investigated the molecular aspects and functional consequences of the interaction between Bak and peroxisomal testis-specific 1 (Pxt1), a noncanonical BH3-only protein exclusively expressed in the testis. Together with various biochemical approaches, this interaction was verified and analyzed at the atomic level by determining the crystal structure of the Bak–Pxt1 BH3 complex. In-depth biochemical and cellular analyses demonstrated that Pxt1 functions as a Bak-activating proapoptotic factor, and its BH3 domain, which mediates direct intermolecular interaction with Bak, plays a critical role in triggering apoptosis. Therefore, this study provides a molecular basis for the Pxt1-mediated novel pathway for the activation of apoptosis and expands our understanding of the cell death signaling coordinated by diverse BH3 domain-containing proteins.

## Abstract 2

Bak is a critical executor of apoptosis belonging to the Bcl-2 protein family. This study uses structural, biochemical, and cellular analyses to reveal how Pxt1, a male germ cell-specific protein, recognizes Bak to activate its pro-apoptotic activity and induce cell death.

## Introduction

B cell lymphoma-2 (Bcl-2) antagonist/killer (Bak) is a critical executor of mitochondrial outer membrane permeabilization (MOMP), the key process of the type I programmed cell death called apoptosis [1–3]. Bak is a member of the Bcl-2 protein family, which comprises 3 subgroups: proapoptotic BH3-only proteins, antiapoptotic Bcl-2 homologues, and downstream MOMP executors Bak and Bcl-2-associated X (Bax) [4,5]. The 3 Bcl-2 family subgroups are intimately associated with each other, which is pivotal for precise regulation of apoptosis. The BH3 domains of Bim, Bid, and Puma, which are called “promiscuous” BH3-only activators, directly interact with the antiapoptotic Bcl-2 homologues [6–8] and also with proapoptotic Bak and Bax [9–13], commonly by being accommodated into a characterized hydrophobic cleft known as the BH3-binding groove. Intermolecular binding of the BH3 domains of Bim, Bid, and Puma initiates the activation of Bak and Bax [1–3]. Once activated, Bak and Bax undergo a conformational change to oligomerize on the mitochondrial membrane, which causes MOMP to be characterized by loss of mitochondrial membrane potential (ΔΨm) and release of cytochrome *c* [14,15]. The released cytochrome *c* triggers the assembly of the apoptosome, a large caspase-activating protein complex, which results in consecutive proteolytic cleavage of caspase proteins and eventual apoptotic cell death [16,17]. In contrast, “selective” BH3-only sensitizers, such as Bad and Noxa, interact with antiapoptotic Bcl-2 relatives but not with Bak and Bax [7,18–21]. In addition, a variety of proteins, which have not been classified as conventional Bcl-2 family members, contain their own BH3 domains that interact with the BH3-binding groove of antiapoptotic Bcl-2 proteins [22,23]. These proteins include the autophagy regulator Beclin 1 [24], necrosis regulator SOUL [25], prosurvival protein TCTP [26], and apoptosis-associated protein peroxisomal testis-specific 1 (Pxt1) [27].

Pxt1, a male germ cell–specific protein exclusively expressed during spermatogenesis [28], was reported to contain a BH3 domain and to induce germ cell apoptosis and male mouse infertility upon overexpression [29]. Recently, we determined the crystal structure of Bcl-xL bound to the BH3 domain of human Pxt1 and revealed that human and mouse Pxt1 differ in terms of amino acid length, BH3 domain composition, and binding ability to Bcl-xL [27]. Furthermore, Aguilar and colleagues reported that the BH3 domain of Pxt1 can directly interact with Bak and induce its activation, which was verified by detecting liposomal membrane permeabilization and cellular cytochrome *c* release [30]. In this study, we present our efforts to unravel the functionality of Pxt1 in apoptosis, which led to the confirmation of a direct interaction between Bak and Pxt1 by a combination of diverse biochemical approaches and the complex structure determination by X-ray crystallography. Subsequent crystal structure-based biochemical and cellular analyses demonstrated that Pxt1 functions as an apoptogenic factor by activating Bak via its BH3 domain-mediated intermolecular association. We demonstrated that Pxt1 not only induces dimerization and activates the liposomal membrane-permeabilizing ability of recombinant Bak, but also causes loss of ΔΨm, release of mitochondrial cytochrome *c* to the cytosol, and significant promotion of apoptotic cell death in its BH3 domain-dependent manner when expressed in cells. Therefore, this study expands our understanding of apoptosis pathways, in which not only conventional Bcl-2 family proteins but also noncanonical members also participate.

## Results

### Human Pxt1 directly interacts with Bax or Bak through its BH3 domain

**Fig 1:**
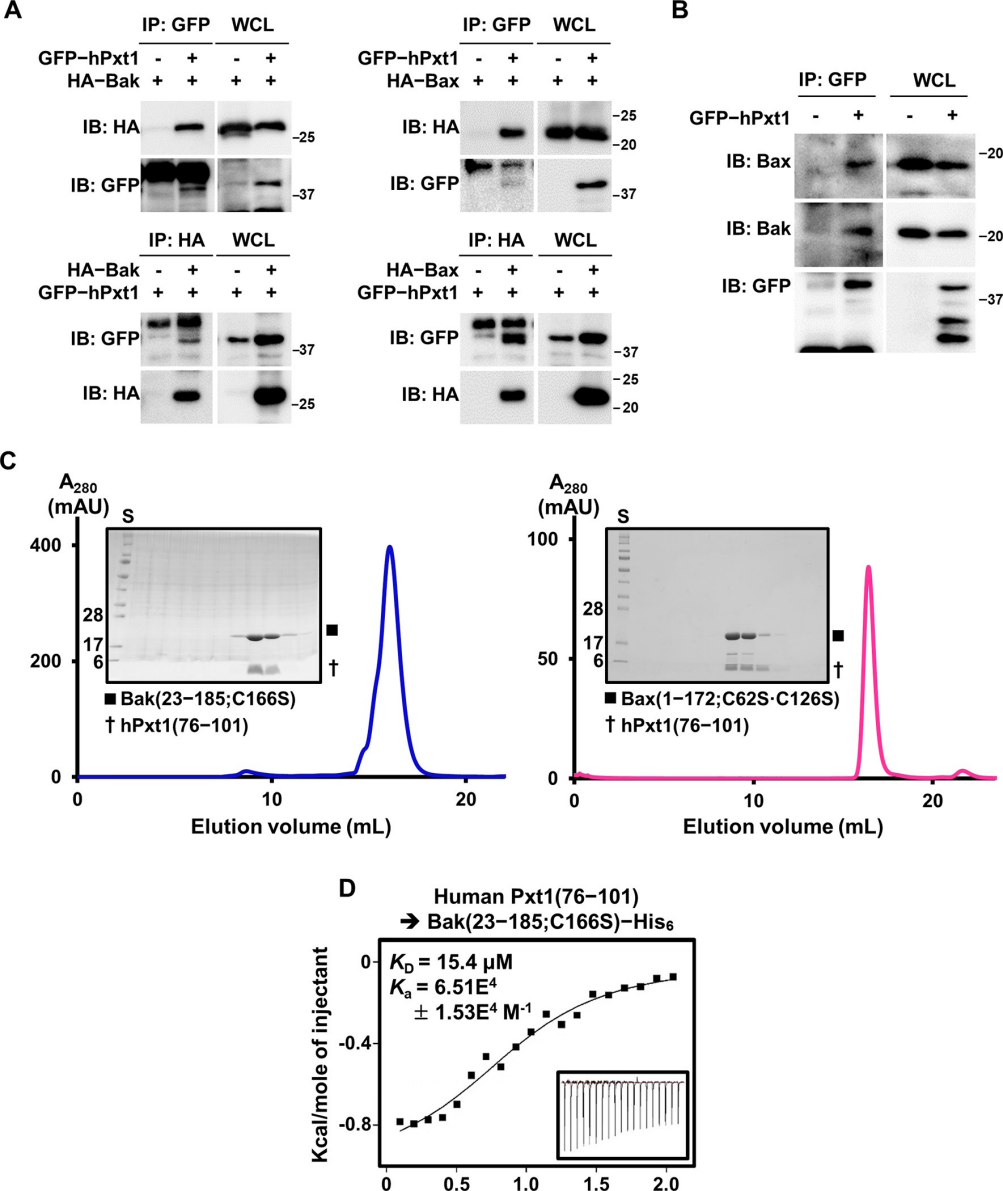
Biochemical analysis of the interaction between Pxt1 and Bak or Bax. (**A**, **B**) Co-immunoprecipitation assay. Intermolecular binding of full-length human Pxt1 to transiently expressed (**A**) or endogenous (**B**) Bak or Bax in HeLa cells was analyzed by immunoprecipitation and immunoblotting. All cells were treated with 20 μM z-VAD-fmk before transfection of plasmids. Full gel figures are available in S1 Raw Images. (**C**) SEC analysis. Recombinant Bak and the indicated human Pxt1 BH3 fragment were copurified and subjected to Superdex 200 increase 10/300 GL column (left). Separately purified recombinant Bax and the Pxt1 BH3 fragment were also mixed and incubated for 1 h, which were then subjected to the same column (right). The peak fractions were analyzed and visualized by SDS–PAGE and Coomassie staining. Full gel figures are available in S1 Raw Images. (**D**) The interaction between recombinant Bak and human Pxt1 BH3 was analyzed by ITC. The numerical data are included in S1 Data. Bak, Bcl-2 antagonist/killer; Bax, Bcl-2-associated X; hPxt1, human Pxt1; ITC, isothermal titration calorimetry; PAGE, polyacrylamide gel electrophoresis; Pxt1, peroxisomal testis-specific 1; S, size marker; SDS, sodium dodecyl sulfate; SEC, size-exclusion chromatography.

Similar to the BH3 domain of Bim and Bid [31,32], Pxt1 BH3 promiscuously interacts with all 5 antiapoptotic Bcl-2 homologues [22]. Given that Bim/Bid BH3 binds the proapoptotic Bcl-2 family regulators Bak and Bax directly [9–13,21,33], we questioned whether Pxt1 BH3 can associate with Bak and Bax as well. We first conducted a co-immunoprecipitation assay to analyze the interaction between full-length Pxt1 and Bak or Bax. When expressed in HeLa cells, human Pxt1 was verified to form a complex with transiently expressed (Fig 1A) or endogenous Bak/Bax (Fig 1B). In contrast, mouse Pxt1, which has a short and incomplete BH3 domain (S1 Fig) and is unable to bind Bcl-xL [27], did not interact with Bak or Bax (S2A Fig). Next, we attempted to confirm their intermolecular association using recombinant proteins. C-terminal His_6_-tagged Bak (residues 23–185;C166S) and N-terminal His_10_–maltose binding protein (MBP)-tagged human Pxt1 BH3 (residues 76–101) were subjected to size-exclusion chromatography (SEC) either separately (S3A Fig, samples 1 and 2) or after mixing at a ratio of 1:3 (S3A Fig, sample 3). S3A Fig shows that the recombinant Bak protein eluted together with human Pxt1 BH3 when the 2 proteins were mixed, suggesting their potential interaction. We also attempted to purify the complex by mixing the cell lysates separately expressing N-terminal His_10_-tagged Bak(23–185;C166S) and His_10_–MBP–human Pxt1(76–101). After Ni–nitrilotriacetic acid (NTA) affinity chromatography, the eluted protein sample was treated with TEV protease to cleave N-terminal His_10_ from Bak(23–185;C166S) and His_10_–MBP from human Pxt1(76–101), followed by the second round of Ni-NTA affinity chromatography and subsequent SEC. Sodium dodecyl sulfate (SDS)-polyacrylamide gel electrophoresis (PAGE) analysis showed that Bak(23–185;C166S) and human Pxt1(76–101) eluted together, further supporting their complex formation (Fig 1C, left). The binding affinity between recombinant Bak and the human Pxt1(76–101) peptide was measured by isothermal titration calorimetry (ITC) analysis; as a result, their dissociation constant (*K*_D_) was quantified as 15.4 μM (Fig 1D). In contrast, the intermolecular interaction of Bak with the mouse Pxt1(1–18) peptide was not detected by ITC (S3B Fig), which was consistent with the immunoprecipitation data (S2A Fig). Similarly, the intermolecular association between Bax and the human Pxt1 BH3 domain was confirmed by a similar complex purification trial, in which separately purified recombinant Bax (residues 1–172;C62S·C126S) and human Pxt1(76–101) were mixed at a molar ratio of 1:3 and then subjected to SEC (Fig 1C, right). Notably, detergents such as 3-[(3-cholamidopropyl)dimethylammonio]-1-propanesulfonate (CHAPS), which was critically necessary for the formation of the 2:2 complex between Bak or Bax and the wild-type Bim or Bid BH3 domain-containing peptides [9,11,12,34], were not included during our complex purification (Fig 1C) and ITC analysis (Fig 1D). Collectively, our biochemical data demonstrated that human Pxt1 directly interacts with Bax or Bak through its BH3 domain, similar to the corresponding domains of Bim or Bid, and this complex formation does not require any detergent such as CHAPS.

### Structure determination of Bak in a complex with human Pxt1 BH3

**Fig 2:**
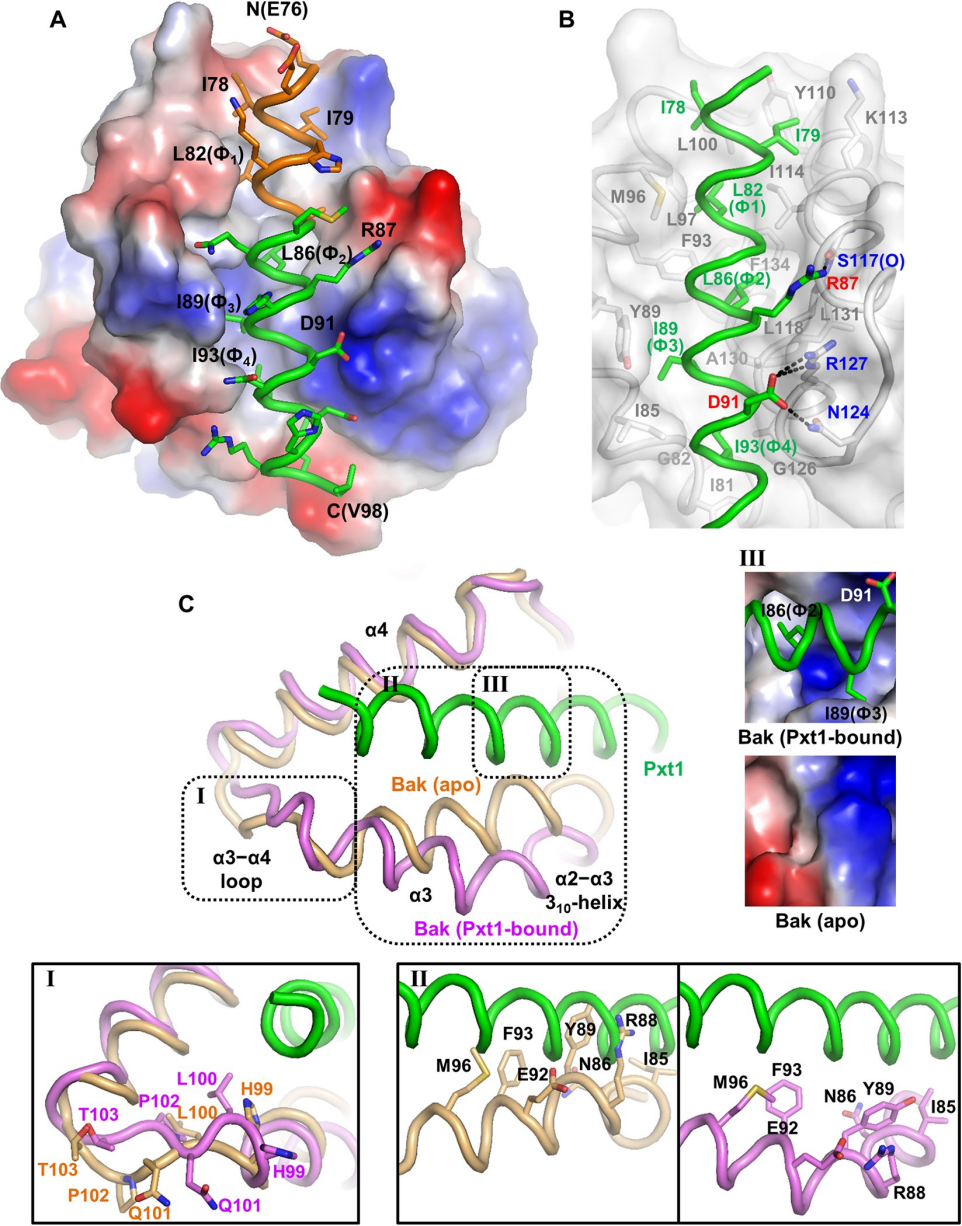
Structural analysis of the interaction between Bak and Pxt1. (**A**) Structural representation. Bak is shown in electrostatic surface representation bound to the human Pxt1 BH3 fragment whose side chains are presented as sticks. Human Pxt1 is shown in green, except for the region absent in mouse Pxt1 that is shown in orange. The Pxt1 residues involved in the intermolecular interaction with Bak are labeled. (**B**) Intermolecular interactions. Pxt1 and Bak are shown in green and gray, respectively. Residues involved in the intermolecular interactions are presented as sticks with labels. Electrostatic interactions and hydrogen bonds between Arg87 and Asp91 of Pxt1 (red) and Ser117, Asn124, and Arg127 of Bak (blue) are marked with dashed lines. (**C**) Structural rearrangement of Bak induced by Pxt1 binding. Pxt1 is shown in green, while Bak is shown in wheat (apo; PDB code 2IMT) or violet (Pxt1-bound form). The Bak regions undergoing prominent conformational changes are marked by boxes and analyzed in detail. Box I, α3−α4 loop; box II, α2–α3 3_10_-helix and α3 helix; box III, a cavity region in the middle of the BH3-binding groove. The red dot in panel III indicates a water molecule. Bak, Bcl-2 antagonist/killer; Pxt1, peroxisomal testis-specific 1.

Next, Bak(23–185;C166S) bound to human Pxt1(76–101) was subjected to crystallization, leading to structural determination of this complex to a resolution of 2.2 Å (S1 Table). To the best of our knowledge, this is the first crystal structure of Bak/Bax complexed with the BH3 domain of a noncanonical BH3-only protein. In this complex structure, the human Pxt1 BH3 fragment forms an amphipathic α-helix and is accommodated in the BH3-binding groove of the single Bak molecule (Fig 2A), similar to its binding to the corresponding region of Bcl-xL, with the equivalent positions of the 5 key BH3 consensus residues (S4A Fig). With the lack of detergent treatment during the purification and crystallization steps, C-terminal helix swapping of Bak was not observed in our structure, which was reported to be induced by the combination of detergent and the BH3 fragment and necessary for the 2:2 complex form [9,11,12,34]. The intermolecular binding between Bak and Pxt1 was mainly mediated by hydrophobic interactions: 4 hydrophobic BH3 consensus residues (Leu82, Leu86, Ile89, and Ile93) and 2 additional nonconserved isoleucine residues (Ile78 and Ile79) of human Pxt1 associate with Ile81, Gly82, Ile85, Tyr89, Phe93, Met96, Leu97, Leu100, Tyr110, Lys113, Ile114, Gly126, Val129, Ala130, Leu131, and Phe134 of Bak (Fig 2B). Based on the complex structure, we introduced alanine substitutions at Leu82 and Leu86 in human Pxt1, referred to as DLA in this manuscript, which were expected to abrogate their intermolecular association. A co-immunoprecipitation assay (S2B Fig) and ITC analyses (S3B Fig) demonstrated that these mutations critically impaired complex formation between Bak and human Pxt1, demonstrating the relevance of the crystal structure. In addition, Ile78, Ile79, and Leu82 of human Pxt1, involved in the intermolecular hydrophobic interaction with Bak, are included in the initial part of the α-helix, which is absent in mouse Pxt1 (Figs 2A and S1). Moreover, this region covers approximately 31.0% (367 Å^2^ of 1,183 Å^2^) of the total buried surface area of Bak with human Pxt1(76–101) in the complex structure (Fig 2A), which could be the reason that mouse Pxt1 does not interact with Bak or Bax. Consistently, the mouse Pxt1(1–18)-corresponding human Pxt1(84–101) peptide did not show a noticeable association with Bak in ITC analysis (S3B Fig). We also found that Asp91, another conserved BH3 consensus residue in human Pxt1, contributes to complex formation by mediating electrostatic interactions with Arg127 and hydrogen bonding with Asn124 of Bak (Fig 2B). The guanidinium group of Arg87 of Pxt1 also forms a hydrogen bond with the main-chain carbonyl of Ser117 of Bak (Fig 2B).

### Binding of Pxt1 induces conformational change of Bak

Previous structural analyses have revealed that the BH3-binding groove of Bak is closed in the apo form [35,36], suggesting that a conformational change of Bak is inevitable for BH3 domain binding. Thus, we compared our Pxt1 BH3-bound Bak structure to that of apo Bak. Structural superimposition indicated that the 2 structures overlap well with each other with a root mean square deviation value of 1.40 Å over 150 aligned C_α_ atoms. However, severe steric hindrance occurred between the human Pxt1 BH3 fragment and the apo-Bak molecule upon superimposition, in which Ile85, Asn86, Arg88, Tyr89, Glu92, Phe93, and Met96 of Bak were involved (Fig 2C, panel II and S4B Fig). In the complex structure, a 3_10_-helix between α2 and α3 (α2–α3 3_10_-helix; residues 84–88) and the α3 helix (residues 89–100) of Bak containing the steric hindrance-associated residues are moved away toward the opposite side of α4, which widens the BH3-binding groove of Bak and enables the human Pxt1 BH3 domain to be accommodated in the pocket (Figs 2C and S4B). Furthermore, binding of human Pxt1 BH3 induces the formation of a “cavity” in the middle of the BH3-binding groove of Bak (Fig 2C, panel III), which was previously reported to reflect the conformational change of Bak or Bax induced by BH3 domain binding [9,11]. We note that the α3–α4 loop region also undergoes noticeable structural rearrangement upon Pxt1 binding (Fig 2C, panel I).

### Structural comparison with other Bak–BH3 complex structures

**Fig 3:**
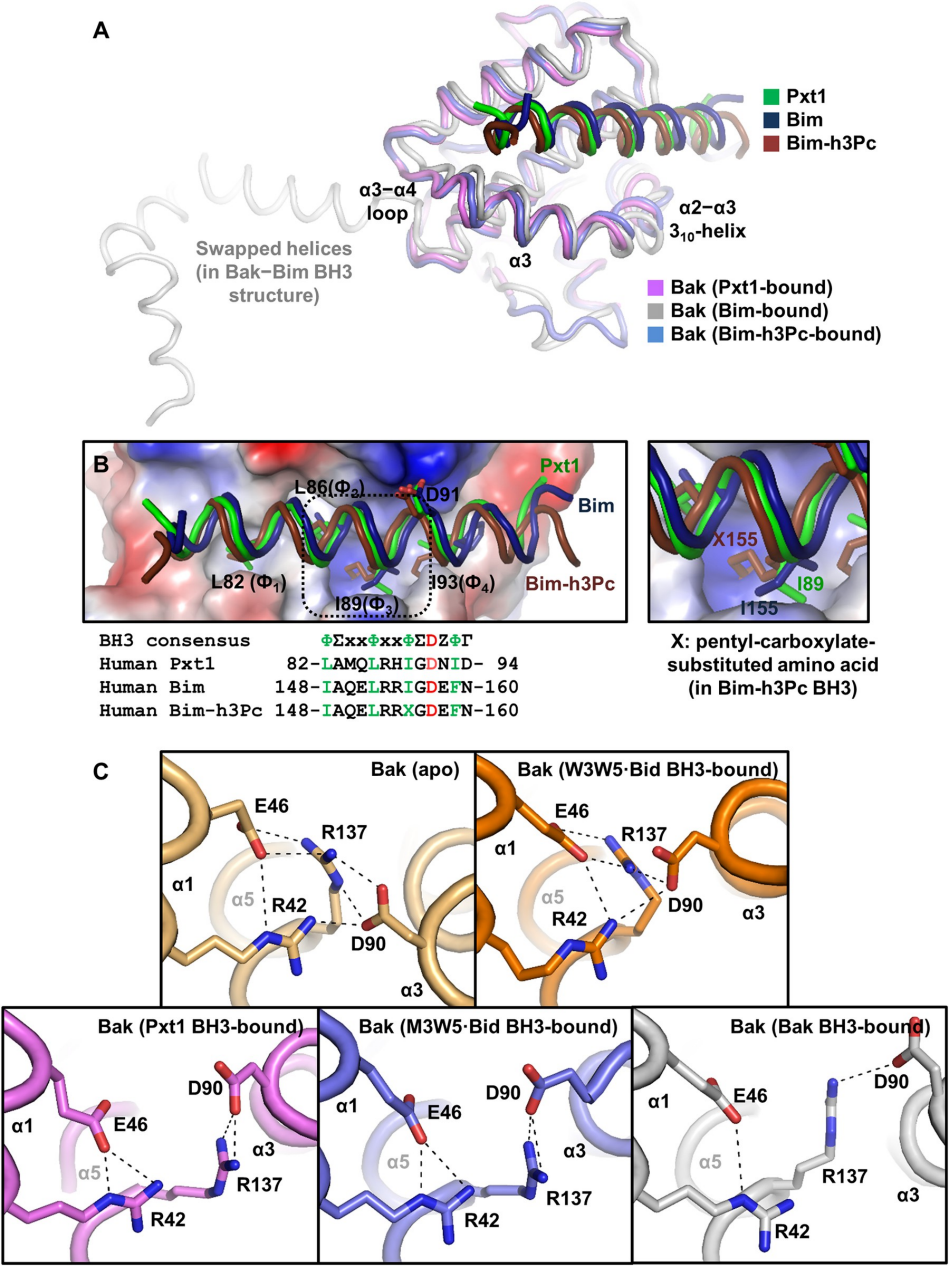
Structural comparison between Bak bound to various BH3 domains. (**A**) Structural alignments of 3 different BH3 domain-bound Bak molecules. The PDB codes are 5VWV for Bak bound to Bim and 5VWZ for Bak bound to Bim-h3Pc. (**B**) Details of the intermolecular interactions. Bak in a complex with Pxt1 is shown in an electrostatic surface representation, and 3 peptides accommodated in the hydrophobic groove region are shown together. The 5 BH3 consensus residues are shown as labeled sticks (top) and aligned (bottom). Ф, hydrophobic residue; Σ, small residue; Z, acidic residue; Γ, hydrophilic residue. The cavity region in the middle of the BH3-binding groove of Bak is marked by a rectangle (left) and shown in a separate box (right). (**C**) Structural comparison of intramolecular electrostatic networks of Bak in the apo or indicated BH3-bound forms. The 4 Bak residues mainly involved in the network are shown as labeled sticks. Dashed lines represent electrostatic interactions. The PDB codes are 2IMT for apo Bak, 7M5A for Bak bound to W3W5-mutant Bid BH3, 7M5B for Bak bound to M3W5-mutant Bid BH3, and 7M5C for Bak bound to Bak BH3. Bak, Bcl-2 antagonist/killer; Pxt1, peroxisomal testis-specific 1.

To date, several molecular structures of Bak–BH3 complexes have been reported, including a modified Bid BH3-bound 1:1 form [10], mutated Bid BH3-bound 1:1 form [13], modified Bim BH3-bound 1:1 form, and wild-type Bim BH3-bound 2:2 C-terminal helix-swapped forms [11]. We compared our structure with those of 2 representative Bak–BH3 complexes determined by X-ray crystallography, in which Bak is bound to wild-type Bim BH3, which functions as the Bak activator (2:2 form; PDB code 5VWV), or modified Bim BH3, which serves as the Bak inhibitor (1:1 form; PDB code 5VWZ). As shown in Fig 3A, Pxt1 BH3 binds Bak in nearly the same manner as the other BH3 domains. Noticeably, the Bak region, including the α2–α3 3_10_-helix, α3, and α3–α4 loop, undergoes similar structural rearrangements upon binding of Pxt1 BH3 or the 2 Bim BH3-derived peptides (Fig 3A; also see Fig 2C). Furthermore, the 5 key BH3 consensus residues in the Pxt1 and Bim BH3 domains occupied equivalent positions for binding to the BH3-binding groove of Bak (Fig 3B, left panel). We noted that the cavity region in the middle of the BH3-binding groove of Bak formed upon BH3 binding (see Fig 2C), which is occupied by the nonnatural pentyl-carboxylate group in the Bak-inactivating Bim-h3Pc BH3-bound structure, is empty but for a single water molecule in the Pxt1 or Bim BH3-bound complexes (Fig 3B, right panel; also see Fig 2C, panel III). This implies that Pxt1 BH3 has the potential to trigger a conformational change of Bak to its dimeric form, which is considered to be necessary for Bak activation to provoke apoptosis.

Recently, a study done by Singh and colleagues demonstrated that destabilization of the intramolecular electrostatic network of Bak, which is mainly constituted by Arg42, Glu46, Asp90, and Arg137, serves as a hallmark of BAK activation [13]. Therefore, we analyzed the state of the electrostatic network in the Bak–Pxt1 BH3 structure. This network was tightly organized in the apo or Bak-inactivating W3W5-mutant Bid BH3-bound form of Bak (Fig 3C, top panels). In contrast, because of the movement of the Bak α3 helix (see Fig 2C, panel II) containing Asp90 and the side chain reorientation of Arg42, Glu46, and Arg137, the network was considerably destabilized in Pxt1 BH3-bound Bak, similar to that in the Bak-activating M3W5-mutant Bid BH3 or Bak BH3-bound form (Fig 3C, bottom panels). This structural analysis supports the notion that Pxt1 activates Bak via direct binding.

### Pxt1 is able to activate recombinant Bak

**Fig 4:**
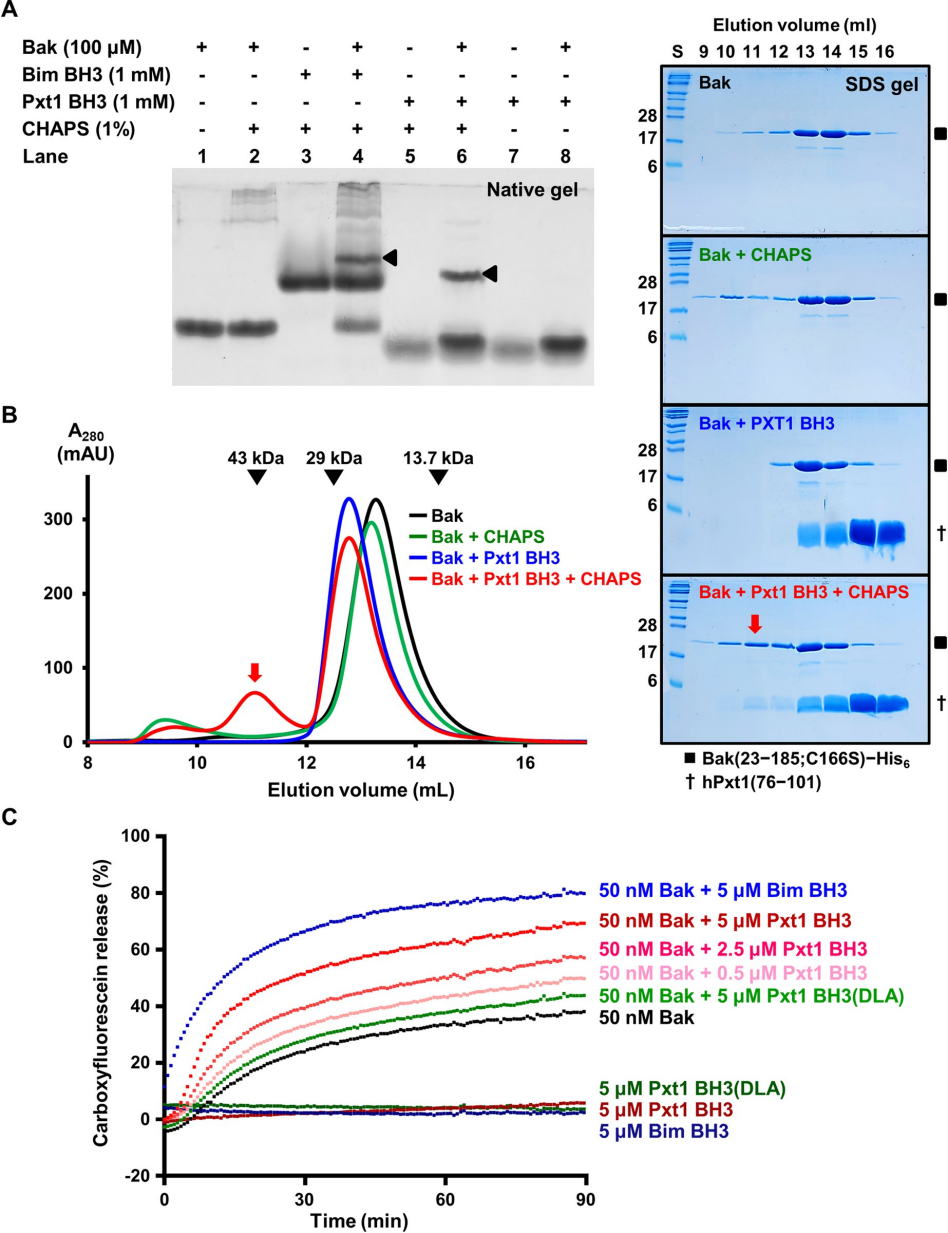
Pxt1 BH3 induces dimerization and activation of recombinant Bak. (**A**) Native gel electrophoresis. Recombinant Bak was incubated with the Bim or Pxt1 BH3 peptide with or without CHAPS. Appearance of a novel protein band can be observed in lanes 4 (100 μM Bak + 1 mM Bim BH3 + 1% CHAPS) and 6 (100 μM Bak + 1 mM Pxt1 BH3 + 1% CHAPS). A full gel figure is available in S1 Raw Images. (**B**) SEC analysis using a Superdex 75 10/300 GL gel filtration column. The elution positions of the standard protein size markers ovalbumin (43 kDa), carbonic anhydrase (29 kDa), and ribonuclease A (13.7 kDa) are indicated by arrowheads. Samples are prepared with the same concentration described in (**A**). The peak fractions from the mixture elution were analyzed and visualized by SDS-PAGE and Coomassie staining, shown on the left. Red arrows indicate the novel peak portion that appeared upon incubation with 100 μM Bak, 1 mM Pxt1 BH3, and 1% CHAPS. Full gel figures are available in S1 Raw Images. (**C**) Liposome release assay. Liposomes encapsulating self-quenching fluorescent dye were incubated with recombinant Bak and/or the indicated BH3 peptide. Release was normalized to detergent-solubilized liposomes. Experiments were performed in independent triplicate, and the numerical data are included in S1 Data. Bak, Bcl-2 antagonist/killer; CHAPS, 3-[(3-cholamidopropyl)dimethylammonio]-1-propanesulfonate; PAGE, polyacrylamide gel electrophoresis; Pxt1, peroxisomal testis-specific 1; S, size marker; SDS, sodium dodecyl sulfate; SEC, size-exclusion chromatography.

Whether Pxt1 indeed triggers the dimerization and activation of Bak was our next question. To answer this issue, we incubated recombinant Bak with Bim/Pxt1 BH3 at a 1:10 molar ratio in the presence or absence of 1% CHAPS for 1 h and subjected the samples to native gel electrophoresis. Fig 4A shows that new bands with a distinctly increased molecular weight appeared when Bak was incubated with Bim/Pxt1 BH3 in the presence of CHAPS (lanes 4 and 6) but not when either BH3 (lane 2) or CHAPS (lane 8) was absent. To further elucidate these results, SEC analysis was performed, which was used by Brouwer and colleagues to demonstrate that the Bim, Bid, and Bak peptides can induce dimerization of Bak upon treatment with CHAPS [11,34]. Similar to previous results, we noticed that a considerable portion of the Bak protein sample was shifted to a new peak region that appeared only in the presence of Pxt1 BH3 and CHAPS, indicating that Pxt1 BH3 can induce dimerization of Bak (Fig 4B).

Next, we conducted a liposomal permeabilization assay, which has been widely used to analyze the Bak-activating capability of the BH3 domain-containing fragments [11,30,37–39]. In the mitochondrial outer membrane–mimicking minimal model liposome system (see Materials and methods), the Bak-dependent release of liposome-entrapped carboxyfluorescein was remarkably enhanced by treatment with Pxt1 BH3 in the dose-dependent manner, demonstrating the ability of Pxt1 BH3 to activate Bak (Figs 4C and S5). In contrast, the Pxt1 BH3 peptide lost its liposome-permeabilizing activity upon introduction of the DLA mutation (Figs 4C and S5), indicating that Pxt1 activity relies on its binding to Bak. Collectively, these results demonstrate that human Pxt1 can induce dimerization and activation of recombinant Bak via its BH3 domain-mediated direct binding.

### Pxt1 controls the permeability of the mitochondrial membrane

**Fig 5:**
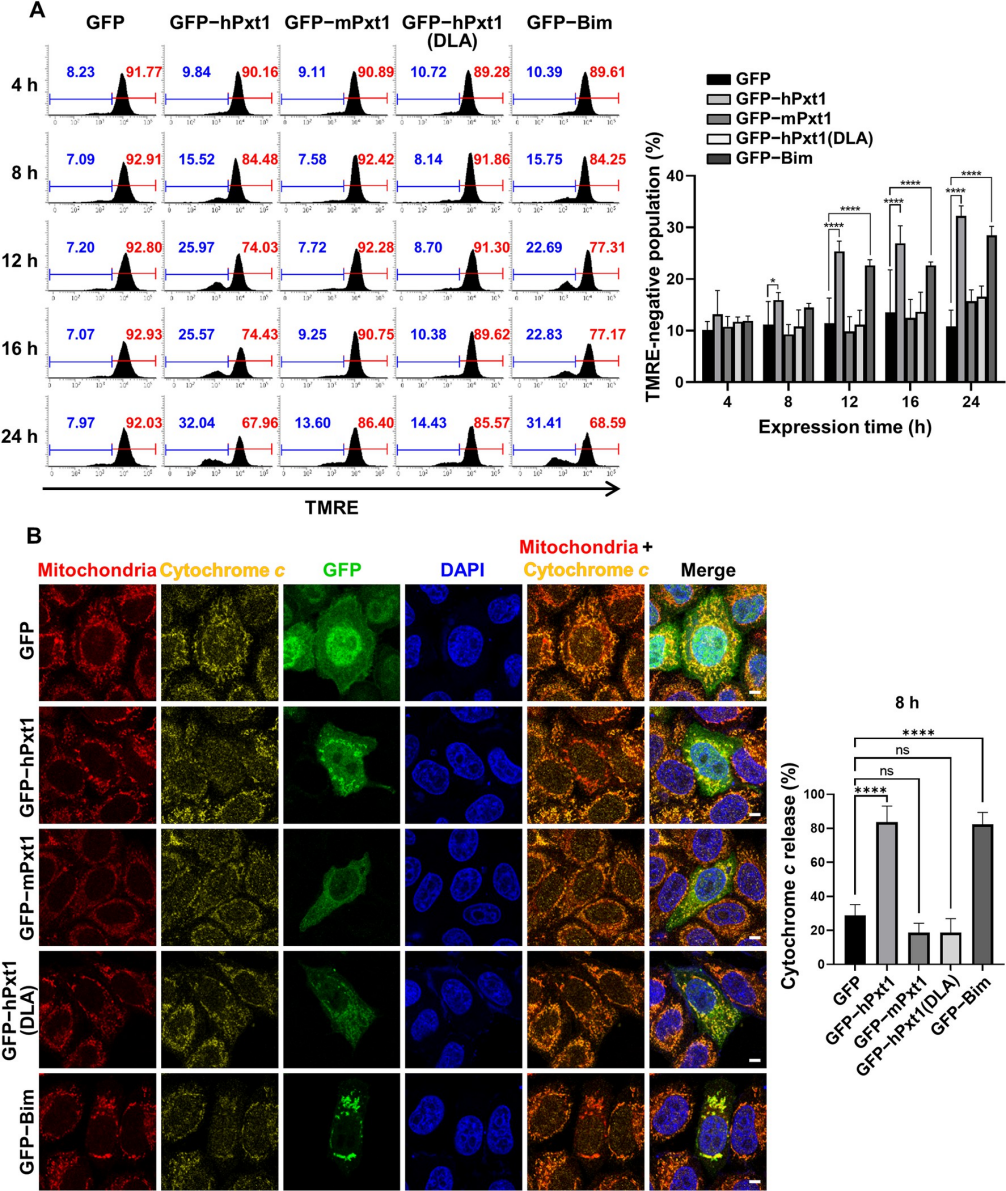
hPxt1 promotes the process of MOMP. HeLa cells transiently expressing the indicated full-length constructs were used for MOMP analysis. (**A**) ΔΨm analysis using TMRE staining followed by flow cytometry. Numbers indicate the percentages of TMRE-negative (blue) or positive (red) mitochondrial populations at each designated hour (left) and are represented as graphs (right). Experiments were performed in 7 replicates *, *P* < 0.05; ****, *P* < 0.001 in the two-way ANOVA followed by Tukey’s HSD. The numerical data are included in S1 Data. The gating strategies of flow cytometry are included in S3A Data. (**B**) Localization of cytochrome *c* was analyzed using confocal microscopy. Mitochondria, cytochrome *c*, GFP, and DAPI were immunostained and visualized in HeLa cells fixed with 4% paraformaldehyde 8 h after transfection. The scale bars indicate 10 μm. The ratio of cells releasing cytochrome *c* upon expression of GFP only (control) or each of the indicated constructs was shown on the right. For each construct, 100–119 cells were counted, which were performed in 3 replicates. ns, nonsignificant; ****, *P* < 0.0001 in the one-way ANOVA followed by Tukey’s HSD. All cells were treated with 20 μM z-VAD-fmk before transfection of plasmids. The numerical data are included in S1 Data. ANOVA, analysis of variance; DAPI, 4′,6-diamidino-2-phenylindole; DLA, alanine substitutions at Leu82 and Leu86; GFP, green fluorescent protein; hPxt1, human Pxt1; HSD, honestly significant difference; MOMP, mitochondrial outer membrane permeabilization; mPxt1, mouse Pxt1; Pxt1, peroxisomal testis-specific 1; TMRE, tetramethylrhodamine ethyl ester; ΔΨm, mitochondrial membrane potential.

As Pxt1 BH3 was verified to activate the liposome-permeabilizing ability of recombinant Bak (Fig 4C), we subsequently questioned the effects of the expression of full-length Pxt1 constructs on the cellular condition of the mitochondria. Therefore, we investigated whether Pxt1 expression causes loss of ΔΨm and release of cytochrome *c* into the cytosol, the 2 representative consequences of Bak activation in the mitochondria reflecting the progression of MOMP [40]. First, the ratio of active mitochondria in HeLa cells was measured by flow cytometry using tetramethylrhodamine ethyl ester (TMRE), a cationic dye that accumulates in the mitochondrial matrix and is used as a ΔΨm indicator [41]. As shown in Fig 5A, the TMRE-negative mitochondrial population was significantly enhanced by the expression of human Pxt1 as well by that of the proapoptotic protein Bim, which was clearly detected at 8–24 h posttransfection. In contrast, mouse Pxt1 and human Pxt1(DLA) were ineffective in collapsing ΔΨm (Fig 5A). Next, confocal microscopic visualization was conducted to analyze whether Pxt1 expression triggered mitochondrial release of cytochrome *c*. The ratio of HeLa cells releasing cytochrome *c* into the cytoplasm, which was less than 30% upon expression of control green fluorescent protein (GFP; 28.9%), reached more than 80% when either GFP-tagged human Pxt1 (83.7%) or Bim (82.3%) was expressed for 8 h (Fig 5B). In contrast, neither mouse Pxt1 (18.8%) nor human Pxt1(DLA; 18.7%) was effective in inducing the cytochrome *c* release in the same condition (Fig 5B). In conclusion, human Pxt1 was verified to permeabilize the mitochondrial membrane in its BH3 domain-dependent manner.

### Pxt1 provokes apoptotic cell death

**Fig 6:**
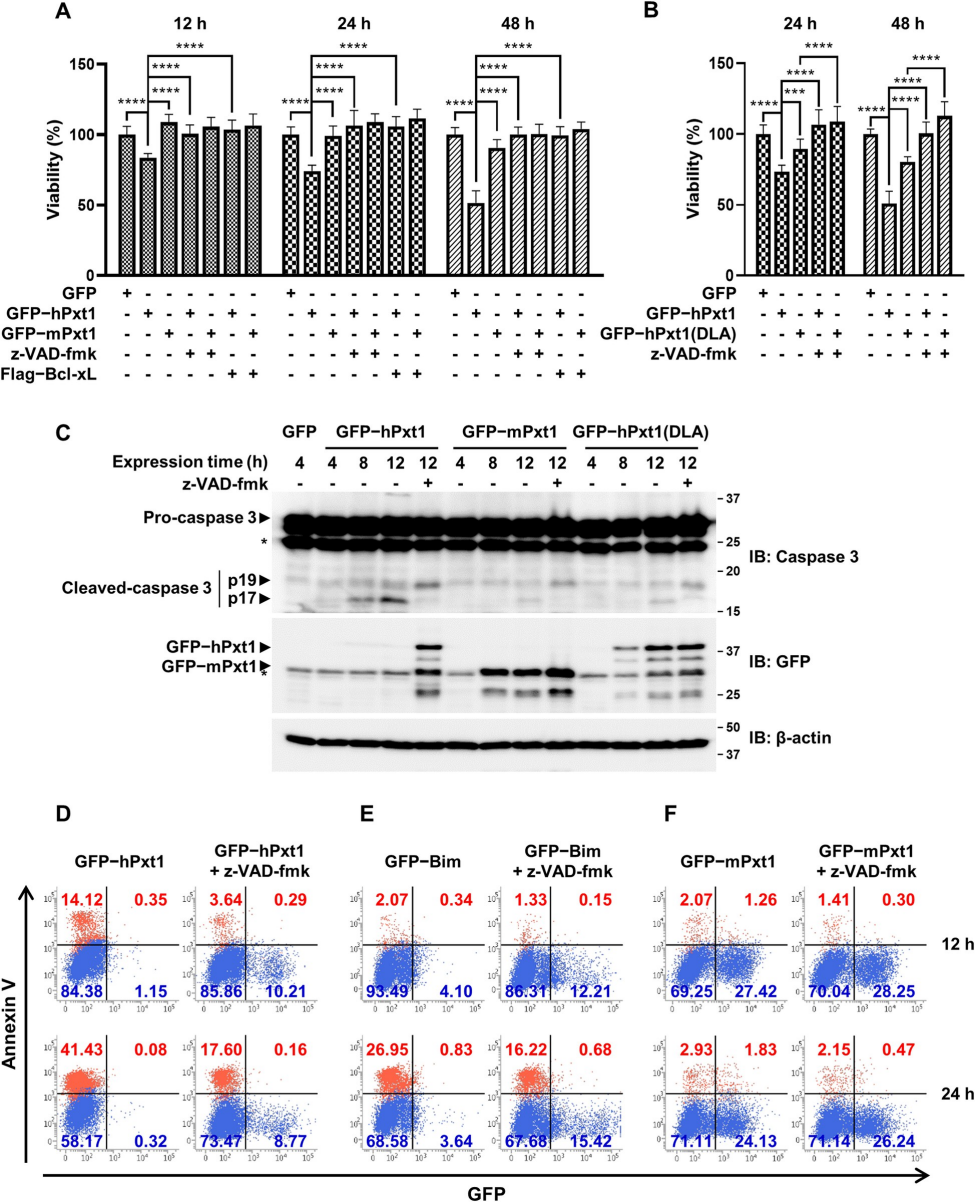
hPxt1 serves as an apoptogenic factor. HeLa cells transiently expressing the indicated full-length constructs were subjected to a cell death analysis. z-VAD-fmk was used to inhibit apoptosis. (**A**, **B**) Cell viability was measured using CellTiter-Glo assay. The effect of human Pxt1 expression on cell viability was compared to that of mouse Pxt1 (**A**) or the Bak binding-defective mutant form (**B**). Experiments were performed in 12 replicates ***, *P* < 0.001; ****, *P* < 0.0001 in the two-way ANOVA followed by Tukey’s HSD. The numerical data are included in S1 Data. (**C**) Cleavage of Pro-caspase 3 was analyzed by immunoblotting after transfection of indicated Pxt1 constructs. *, nonspecific band. Full gel figures are available in S1 Raw Images. (**D**–**F**) Annexin V staining was analyzed by flow cytometry. HeLa cells expressing GFP-tagged hPxt1 (**D**), Bim (**E**), or mouse Pxt1 (**F**) were stained with APC-conjugated annexin V for 5 min, and sorted at 12 or 24 h posttransfection. In each quadrant, the ratio of cells positive (red) and negative (blue) for annexin V is shown at the top and bottom, and those positive and negative for GFP are shown on the right and left, respectively. The gating strategies of flow cytometry are included in S3B Data. ANOVA, analysis of variance; APC, allophycocyanin; Bak, Bcl-2 antagonist/killer; DLA, alanine substitutions at Leu82 and Leu86; GFP, green fluorescent protein; hPxt1, human Pxt1; HSD, honestly significant difference; mPxt1, mouse Pxt1; Pxt1, peroxisomal testis-specific 1.

As our data clearly showed that Pxt1 operates similarly to the BH3-only activators Bim and Bid, it was tempting to speculate that Pxt1 functions as an apoptogenic factor. Prior to the investigation, we checked the protein levels when HeLa cells were transfected with a human or mouse Pxt1 expression vector. Unexpectedly, we could not detect any GFP-tagged protein in cells transfected with the GFP-tagged human Pxt1-expressing vector (S6A Fig; left panel, lane 2). This was not due to proteasomal degradation, as treatment with the proteasome inhibitor MG132 did not result in the recovery of the human Pxt1 protein level (S6A Fig; left panel, lane 4). In contrast, human Pxt1 appeared when cells were treated with the pan-caspase inhibitor z-VAD-fmk (S6A Fig; left panel, lane 3) or when antiapoptotic Bcl-xL was coexpressed (S6A Fig; left panel, lane 5). We note that the expression of human Bim was also hardly detected without z-VAD-fmk treatment (S6B Fig). These results strongly suggest that apoptosis occurred due to the transient expression of human Pxt1 or Bim, which might shield this protein from antibody detection. In contrast, we noticed that expression of mouse Pxt1 and human Pxt1(DLA), which were defective in binding to Bak (S2 and S3B Figs), was easily detected in HeLa cells (S6A Fig; middle and right panels) and hardly affected by apoptosis inhibition (S6A Fig; middle and right panels). Next, viability analysis using a cellular ATP content-based luminescence assay was conducted to analyze the effects of Pxt1 expression on cell death. We found that cell death was triggered in a time-dependent manner by the expression of human Pxt1 but not by that of mouse Pxt1 (Fig 6A) or critically weakly by that of human Pxt1(DLA) in HeLa cells (Fig 6B). The cell death–inducing capability of human Pxt1 was comparable to that of Bim, suggesting that Pxt1 serves as an effective proapoptotic factor (S7A Fig). We also noticed that human Pxt1-induced cell death was severely attenuated by the apoptosis inhibitor z-VAD-fmk treatment or antiapoptotic Bcl-xL coexpression (Figs 6A, 6B, and S7A). Moreover, cleavage of Pro-caspase 3 to the cleaved fragments, a characteristic feature of apoptosis, was observed to occur in HeLa cells in the time-dependent manner upon the expression of human Pxt1 or Bim (Figs 6C and S7B). These results demonstrated that human Pxt1 induces cell death via the apoptosis pathway.

The fate of human or mouse Pxt1-expressing HeLa cells was further analyzed using flow cytometry that determines the rate of apoptosis. We found that the ratio of allophycocyanin (APC)-conjugated annexin V-positive apoptotic cells reached 14% at 12 h and 41% at 24 h post human Pxt1 transfection (Fig 6D, left panels), which were higher than those (2% at 12 h and 28% at 24 h) induced by human Bim transfection (Fig 6E, left panels). In contrast, they were suppressed to 4% at 12 h and 18% at 24 h after human Pxt1 transfection (Fig 6D, right panels) and 1% at 12 h and 17% at 24 h after human Bim transfection (Fig 6E, right panels) by z-VAD-fmk treatment. When HeLa cells were transfected with the plasmid expressing mouse Pxt1 instead of human Pxt1, less than 5% of cells were positive for annexin V irrespective of z-VAD-fmk treatment (Fig 6F). We also noticed that the proportion of GFP-positive cells was approximately 2% or 5% after transfection for expression of GFP-tagged human Pxt1 (Fig 6D, left panels) or Bim (Fig 6E, left panels), but it became about 10% with z-VAD-fmk treatment (Fig 6D, right panels) and above 25% upon expression of mouse Pxt1 instead of human Pxt1 (Fig 6E). These results are consistent with the results of the immunoblotting analysis showing that the expression of human Pxt1 or Bim, but not that of mouse Pxt1, was undetectable without apoptosis inhibition (S6A and S6B Fig). Cell sorting analysis using flow cytometry confirmed that human Pxt1(DLA) is less effective than wild-type human Pxt1 or Bim in triggering apoptosis when transiently expressed in HeLa cells (S8 Fig), which was consistent with the results of the luminescence-based apoptosis assay (Fig 6B). Collectively, these results indicate that human Pxt1 functions as an effective proapoptotic factor, and its BH3 domain, which mediates interaction with Bak, plays a key role in its cell death–inducing capability.

### Pxt1 requires proapoptotic executors such as Bak to induce apoptosis

**Fig 7:**
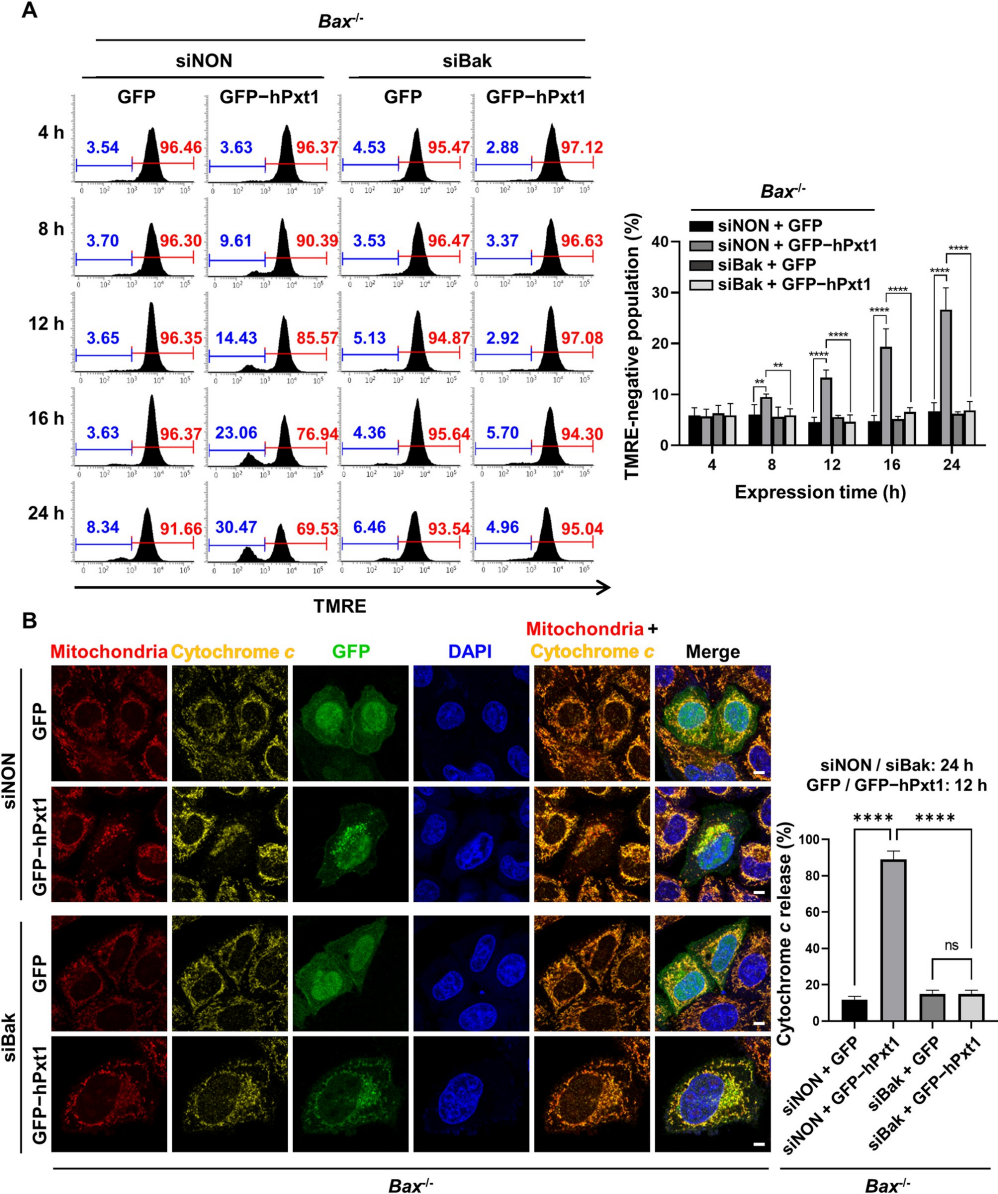
Bak plays a critical role in the hPxt1-mediated MOMP progress. *Bax*^−/−^ HeLa cells transiently expressing control GFP or GFP-tagged full-length hPxt1 were transfected with siNON or siBak, as indicated, and used for MOMP analysis. (**A**) ΔΨm analysis by flow cytometry. Numbers indicate the percentages of TMRE-negative (blue) or positive (red) mitochondrial populations at each examination (left) and are represented as graphs (right). Experiments were performed in 7 replicates. **, *P* < 0.01; ****, *P* < 0.0001 in the two-way ANOVA followed by Tukey’s HSD. The numerical data are included in S1 Data. The gating strategies of flow cytometry are included in S3A Data. (**B**) Confocal microscopy analysis of the release of cytochrome *c*. *Bax*^−/−^ HeLa cells were transfected with siNON or siBak for 24 h, followed by transfection with GFP or GFP−hPxt1-expressing vector for additional 12 h. The mitochondria, cytochrome *c*, GFP, and DAPI were immunostained and visualized fixed with 4% paraformaldehyde posttransfection. The scale bars indicate 10 μm. The ratio of cells releasing cytochrome *c* upon expression of the indicated constructs was shown on the right. For each construct, 53–69 cells were counted, which were performed in 3 replicates. ns, nonsignificant; ****, *P* < 0.0001 in the one-way ANOVA followed by Tukey’s HSD. All cells were treated with 20 μM z-VAD-fmk before transfection of plasmids. The numerical data are included in S1 Data. ANOVA, analysis of variance; Bak, Bcl-2 antagonist/killer; DAPI, 4′,6-diamidino-2-phenylindole; GFP, green fluorescent protein; hPxt1, human Pxt1; HSD, honestly significant difference; MOMP, mitochondrial outer membrane permeabilization; TMRE, tetramethylrhodamine ethyl ester; ΔΨm, mitochondrial membrane potential.

**Fig 8:**
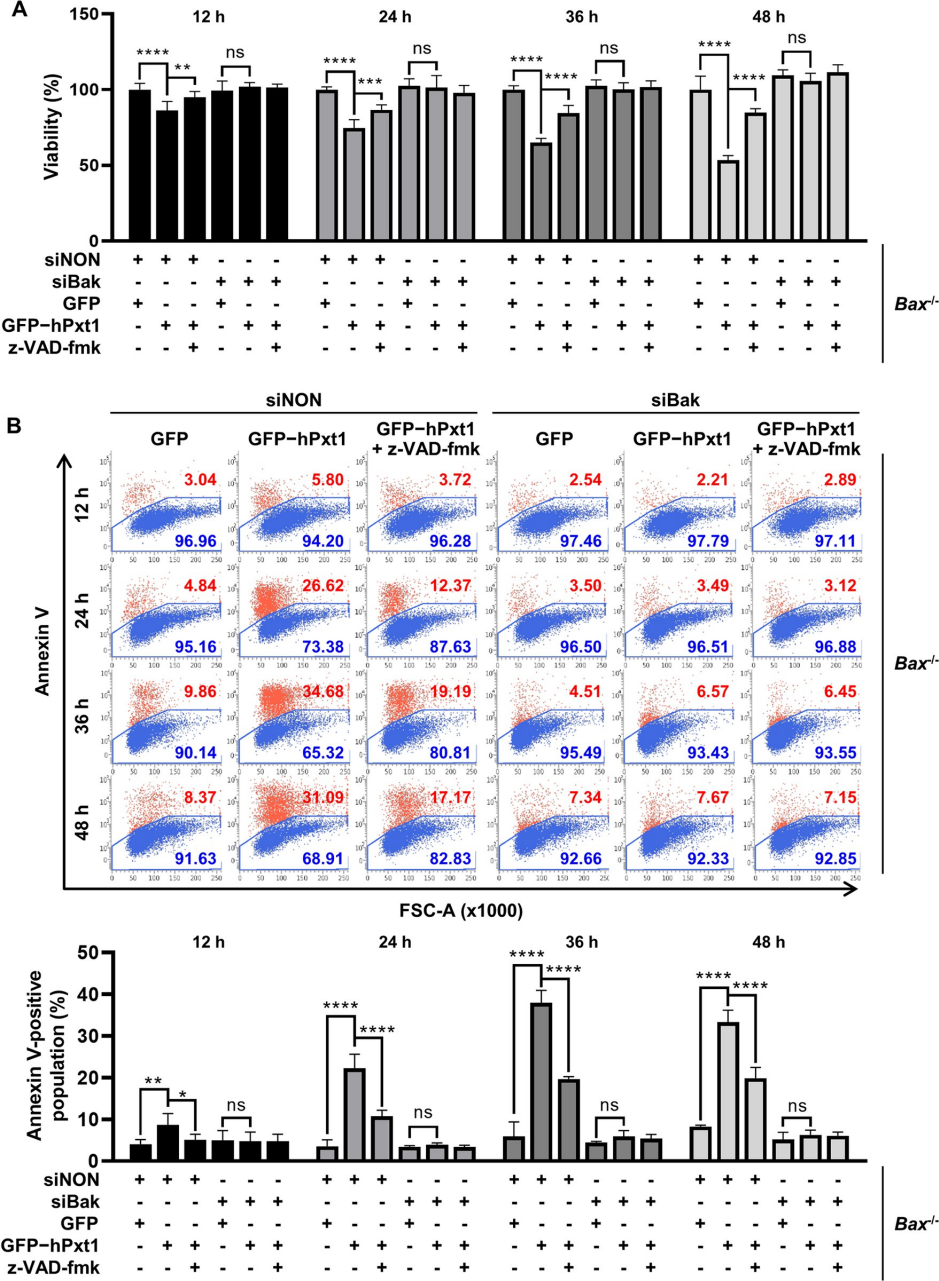
Bak serves as a key factor in hPxt1-mediated apoptosis. *Bax*^−/−^ HeLa cells transiently expressing control GFP or GFP-tagged full-length hPxt1 were treated with siNON or siBak, as indicated, and subjected to cell death analysis. (**A**) Cell viability was analyzed using the CellTiter-Glo assay. Experiments were performed in 9 replicates. ns, nonsignificant; **, *P* < 0.01; ***, *P* < 0.001; ****, *P* < 0.0001 in the two-way ANOVA followed by Tukey’s HSD. The numerical data are included in S1 Data. (**B**) Annexin V staining-based cell death analysis using flow cytometry. HeLa cells in each condition were stained with APC-conjugated annexin V for 5 min and then sorted at the indicated time points posttransfection/treatment. The ratios of cells annexin V–positive and negative cells are shown in red and blue, respectively (top), and are represented as graphs (bottom). Experiments were performed in 7 replicates. ns, nonsignificant; *, *P* < 0.05; **, *P* < 0.01; ***, *P* < 0.001; ****, *P* < 0.0001 in the two-way ANOVA followed by Tukey’s HSD. The numerical data are included in S1 Data. The gating strategies of flow cytometry are included in S3C Data. ANOVA, analysis of variance; APC, allophycocyanin; Bak, Bcl-2 antagonist/killer; FSC-A, forward scatter area; GFP, green fluorescent protein; hPxt1, human Pxt1; HSD, honestly significant difference.

Our results demonstrated that Pxt1, which interacts with (Figs 1–3) and activates (Fig 4) Bak, is a functionally active proapoptotic factor (Figs 5 and 6). Subsequently, we investigated the functional significance of Bak in Pxt1-induced MOMP and apoptosis. To this end, *Bax*-deficient (*Bax*^−/−^) HeLa cell lines were generated using CRISPR (S6C Fig, left). Genetic suppression of Bak expression was achieved by *Bak*-targeting small interfering RNA (siRNA) called siBak (S6C Fig, right). *Bax*^−/−^ HeLa cells were transfected with control siRNA (siNON) or siBak for 48 h and then followed by transfection with the plasmid expressing control GFP or GFP-tagged human Pxt1. We found that human Pxt1 effectively caused destabilization of ΔΨm (Fig 7A, left) in siNON-treated *Bax*^−/−^ HeLa cells, implying that the presence of Bak is sufficient for human Pxt1 to modulate MOMP progression. In contrast, knockdown of Bak by siBak in *Bax*^−/−^ HeLa cells critically abrogated the ability of Pxt1 to induce loss of ΔΨm (Fig 7A, right). Next, the release of cytochrome *c* in *Bax*^−/−^ HeLa cells was examined using a confocal microscope (Fig 7B). Upon transfection of siNON, expression of GFP-tagged human Pxt1 prominently increased the ratio of cytochrome *c*-releasing cells (89%) compared to that of control GFP (12%). However, when siBak instead of siNON was transfected in *Bax*^−/−^ cells, the ratio of cytochrome *c*-releasing cells was suppressed to be 15% irrespective of the expression of control GFP or GFP-tagged human Pxt1, implying that Bak plays a critical role in the release of cytochrome *c* in the genetic ablation of Bax (Fig 7B). Likewise, treatment with siBak was verified to be highly effective in retarding human Pxt1-induced apoptotic death of *Bax*^−/−^ HeLa cells, as was the case with the pan-caspase inhibitor z-VAD-fmk, which was verified by the ATP content-based viability assay (Fig 8A) and annexin V–staining apoptosis assay (Fig 8B). Consistently, expression of human Pxt1 was much more easily detected in *Bax*^−/−^ HeLa cells treated with siBak than those treated with siNON (S6D Fig), additionally supporting that apoptosis prevented this protein from antibody detection (see S6A Fig). Therefore, these data demonstrate that human Pxt1 triggers apoptosis via the mitochondrial destabilization route associated with Bcl-2 family members and that Bak is sufficient for mediating the proapoptotic activity of Pxt1.

## Discussion

### Pxt1, a noncanonical BH3-only protein that interacts with and activates Bak

Activation of Bak and Bax, which results in their conformational conversion from monomer to homomultimer on the mitochondrial membrane and serves as a gate for cytochrome *c* release, is considered to be a critical process in the apoptosis pathway [1–3]. Diverse proteins are known to serve as regulators of Bak and Bax. In fact, most of them are members of the Bcl-2 family, including Bim, Bid, and Puma, which are called BH3-only activators [9–13], Bmf and Hrk, which were recently reported to activate Bak by interacting with an alternative binding site [42], and antiapoptotic Bcl-2 proteins, which recognize the BH3 domain of Bak/Bax by their own hydrophobic groove [31,43–46]. To date, a number of intensive structural studies have improved our understanding of the regulatory mechanism of apoptosis orchestrated by the molecular associations among Bcl-2 family members. In addition, several proteins also have been additionally identified to interact with Bak/Bax, such as dynamin-related protein 1 [47], linker histone H1.2 [48], voltage-dependent anion channel 2 [49,50], and Pxt1 [30]. Nevertheless, none of the interplays between these proteins and the MOMP executors Bak and Bax has been elucidated at the atomic level by three-dimensional structure determination, limiting our understanding of their precise functional mechanism and magnitude of contribution to the apoptotic pathway. In this study, we comprehensively investigated the structural and functional properties of Pxt1, a noncanonical BH3-only protein exclusively expressed in the testis. Human Pxt1 directly interacts with Bak, which was verified by a combination of biochemical assays including a co-immunoprecipitation assay using Pxt1-expressing cell lysates (Fig 1A and 1B), SEC and ITC analyses using recombinant proteins (Figs 1C, 1D, and S3A), and determination of the crystal structure of Bak bound to the BH3 domain of Pxt1 (Fig 2). To the best of our knowledge, this is the first case of structural elucidation of the interaction between Bak/Bax and the BH3 domain of a noncanonical BH3-only protein.

In our crystal structure, the Pxt1 BH3 domain was bound to the BH3-binding groove of Bak via multiple hydrophobic interactions together with electrostatic and hydrogen bonds (Fig 2A and 2B). The canonical BH3 consensus residues, which are conserved as Leu82, Leu86, Ile89, Asp91, and Ile93 in human Pxt1, play a critical role in intermolecular interactions, in an equivalent manner to the binding of typical BH3 peptides (Fig 3). Simultaneously, remarkable differences were observed when our structure was compared to the previously reported canonical Bak–wild-type BH3 complex structures; the association of Pxt1 BH3 with Bak/Bax neither required detergent treatment (Fig 1C and 1D) nor induced C-terminal helix swapping-involved dimerization of Bak (Figs 2 and 3). Instead, binding of the single Pxt1 BH3 fragment induces a conformational change of Bak by itself, which enables its accommodation into the BH3-binding groove of Bak in its monomeric form (Figs 2C, 3C, and S4B). The transient one-to-one association of Bim or Bid initiates the activation of Bak/Bax, followed by their homodimerization and the subsequent formation of their oligomerized pore forms [10,11,13,34,51]. Therefore, we consider that our complex structure represents a “snapshot” of the initial step of the Bak activation process induced by human Pxt1 binding. We confirmed the formation of a “cavity” in the middle of the BH3-binding groove of Bak upon human Pxt1 BH3 binding (Figs 2C and 3B) and the destabilization of the α1 helix-mediated electrostatic network in Bak (Fig 3C), which commonly reflect the conformational change of Bak [11]. Subsequent biochemical analyses demonstrated that Pxt1 induced dimerization (Fig 4A and 4B) and promoted membrane permeabilization activity of the recombinant Bak protein (Fig 4C). Moreover, human Pxt1 triggered MOMP progression (Fig 5) and apoptotic cell death (Figs 6 and S7) in the comparable level with Bim. Similar to BH3-only activators such as Bim/Bid, Pxt1 did not require the support of any additional apoptotic stimulus for its proapoptotic activity (Figs 5 and 6), further supporting the notion that Pxt1 serves as a highly effective apoptogenic factor. The functional association between Bak and Pxt1 was further analyzed using *Bax*-knocked out cells, in which apoptosis should depend on the activity of Bak. At least in this condition, Bak was verified to play a critical role in the proapoptotic functionality of Pxt1, as gene knockdown of Bak severely attenuated MOMP progression and apoptotic cell death of *Bax*^−/−^ HeLa cells despite overexpression of Pxt1 (Figs 7 and 8). Since Bax was shown to interact with Pxt1 (Fig 1), we suppose that Pxt1 can also control apoptosis by targeting Bax for activation, which is our next issue to be investigated. Moreover, we noticed that the apoptogenic functionality of Pxt1 depends on its Bak/Bax-interacting ability via the BH3 domain because both mouse Pxt1 and L82A·L86A mutation-containing human Pxt1, which are both defective in binding Bak/Bax (S1–S3 Figs), showed drastically impaired activity for provoking MOMP and apoptosis (Figs 5, 6, and S8).

### Pxt1 is presumed to control apoptosis by two different pathways

Actually, Bak and Bax are not the unique proteins that bind Pxt1. This protein was previously reported to interact with all 5 antiapoptotic Bcl-2 homologues [22]. Moreover, we recently reported the crystal structure of Bcl-xL bound to Pxt1 BH3 [27]. Therefore, we suppose that Pxt1 is able to promote apoptosis by 2 BH3 domain-associated different pathways like the canonical BH3-only activators: first, by induction of the release of the BH3-only activators from the antiapoptotic Bcl-2 family members such as Bcl-xL, and second, by activation of Bak/Bax via direct binding. Since Pxt1 uses nearly the same residues for interacting with Bcl-xL and Bak (S4A Fig), it appears difficult to experimentally discern the 2 pathways. However, we consider that the interaction with Bax/Bax might play at least an unignorable role in the Pxt1-mediated apoptosis induction. It is because Pxt1 BH3 not only provoked the conformational change of Bak similarly with other Bak-binding activators (Figs 2C and 3C), but also induced dimerization (Fig 4A and 4B) and up-regulated the membrane-permeabilizing activity of Bak (Fig 4C) by itself, even in the absence of other Bcl-2 family members. Even though the Pxt1 BH3-binding affinity was higher in Bcl-xL (*K*_D_ of 233 nM) [27] than in Bak (*K*_D_ of 15.4 μM; Fig 1D) when measured by ITC, we note that it is common among the BH3-only proteins; the ITC-measured *K*_D_ of Bid BH3 for Bcl-xL and for Bak are 8.62 nM [31] and 22.7 μM [13], respectively. Therefore, we hypothesize that Pxt1 BH3 activates Bak by a transient low-affinity binding called the “hit-and-run” interaction, as other BH3-only activators such as Bim and Bid were proposed to do so [10,11,13,34,52–55]. Some other factors such as membrane lipids might facilitate Pxt1 BH3-mediated activation of Bak/Bax, as the case of Bid BH3 [39,56,57].

### Human Pxt1 is a male germ cell–specific apoptogenic factor

While Bim, Bid, and Puma, the 3 conventional BH3-only activators, are ubiquitously expressed in diverse tissues and function as potent inducers of apoptosis, Pxt1 is expressed exclusively in the testis during spermatogenesis and thus appears to play a relatively limited role in controlling apoptosis in the male reproductive system [28]. We hypothesized that male germ cells require additional specific cell death–inducing factor(s) such as Pxt1, because of their extraordinarily high incidence of apoptosis to maintain germline homeostasis by eliminating excessive undifferentiated or progenitor spermatogonia and dysfunctional differentiated spermatocytes and spermatids [58]. Intriguingly, mouse and human germ cells undergo different spermatogenetic processes: While a single mouse spermatogonial stem cell yields 512 differentiated cells through 9 doublings that are divided 3 more times to produce 4,096 round spermatid cells, a single human spermatogonium undergoes only 3 divisions to generate 8 differentiated cells that finally yield 64 spermatids [59]. As a result, the ratio of undifferentiated cells per total germ cells are 0.3% and 22% and sperm outputs per gram of testicular tissue at a single day are 40 million and 4.4 million in mouse and human, respectively [59]. In this study, we found that the BH3 domain of mouse Pxt1 is unable to immunoprecipitate Bak or Bax (S2A Fig), make an interaction with recombinant Bak (S3B Fig), induce mitochondria destabilization (Fig 5), and trigger cell death (Fig 6), unlike the corresponding domain of its human orthologue. At the molecular level, it can be accounted for by the absence of the N-terminal region of human Pxt1 (residues 1–83) in mouse Pxt1 (Figs 2A and S1), which contains Ile78, Ile79, and Leu82 that contribute to the intermolecular association with Bak (Fig 2B). However, a subsequent question remains to be unresolved, which is the evolutionary reason for such functional loss of mouse Pxt1. We assume that it is involved in the aforementioned distinct spermatogenetic processes of mouse and human in which apoptosis should be controlled differently. Since the effects of Pxt1 were analyzed in HeLa cells upon being overexpressed in this study, future researches using male germ cells, in which Pxt1 is endogenously highly expressed, will be helpful to reveal the physiological functionality of human and mouse Pxt1 precisely. We also guess the existence of additional unidentified BH3 domain-containing apoptogenic factors specifically expressed in other tissues, which remains to be elucidated by further investigations.

### Concluding remark

In this study, we delineated the molecular details and functional consequences of the interaction between Bak and human Pxt1, which was determined to be an effective proapoptotic initiator. Therefore, our work expands our understanding of the regulation of Bak and Bax, the critical executors of MOMP and apoptosis. We speculate that Pxt1 is not unique but is the first case of a noncanonical BH3-only protein activating Bak/Bax. Thus, the identification and molecular characterization of unidentified Bak/Bax-interacting factors would be an interesting topic for future research in this field. We also expect that our research will provide a rational basis for therapeutic approaches, especially those involved in controlling apoptosis that occurs during spermatogenesis.

## Materials and methods

### Preparation of proteins, peptides, and chemical

For protein production, we prepared 2 modified pET21a plasmids, pET21a-m1 and pET21a-m2, which produced proteins with an N-terminal (His)_10_ tag and an N-terminal (His)_10_–MBP tag, respectively. The DNA fragment coding for human Bak (residues 23–185;C166S) was cloned into the wild-type pET21a plasmid (Merck, Germany) for biochemical analyses and into the pET21a-m1 plasmid for crystallization. The DNA fragments coding for human Bax (residues 1–172; C62S·C126S) and Pxt1 (residues 76–101) was cloned into the pET21a-m1 and pET21a-m2 plasmids, respectively. Cys-to-Ser mutations were introduced into Bak and Bax constructs based on previous reports [9,11,34]. The recombinant proteins were produced in the *E*. *coli* BL21(DE3) RIL strain (Merck) cultured in Luria-Bertani medium at 25°C for 16 h. Protein purification was conducted using a Ni-NTA column (QIAGEN, Germany) and a HiLoad 26/600 Superdex 75 pg gel filtration column (GE Healthcare) equilibrated with a buffer solution containing 20 mM Tris–HCl (pH 8.0) and 150 mM NaCl. Synthetic peptides derived from mouse Pxt1 (residues 1–18), human Pxt1 (residues 76–101, 76–101 with L82A·L86A mutations, or 84–101), and human Bim (residues 141–166) were purchased from Peptron, Korea. CHAPS was purchased from Merck. ITC measurements using recombinant proteins and synthetic peptides were performed as previously described [27].

### Crystallization and structural determination

For complex formation, human Bak(23–185;C166S) and Pxt1(76–101) were copurified after mixing the cell lysates. Crystals were obtained by the sitting-drop vapor diffusion method at 18°C in which 1 μL protein solution (22 mg/mL) and 1 μL precipitant solution containing 0.1 M sodium citrate (pH 4.8) and 17% (w/v) polyethylene glycol 3000 were mixed and equilibrated. Before the data collection process, the crystals were immersed briefly in a reservoir solution supplemented with 20% glycerol for cryoprotection. Diffraction data were collected on beamline 5C at the Pohang Accelerator Laboratory, Korea, and processed using the program *HKL*2000 [60]. The complex structure was determined by the molecular replacement method with the program Phaser [61], using the structure of Bak bound to Bim-h3Pc with PDB code 5VWZ [11] as a search model. The Coot [62] and PHENIX [63] programs were used for model building and refinement, respectively. The crystallographic data are summarized in S1 Table.

### Liposome assay

C-terminally (His)_6_-tagged recombinant human Bak(23–185;C166S) and synthetic human Pxt1(76–101; wild-type or DLA) or Bim(141–166) peptides were used in the liposome release assay. To mimic the outer mitochondrial membrane, liposomes were prepared by mixing 46% phosphatidylcholine (Merck), 25% phosphatidylethanolamine (Merck), 11% phosphatidylinositol (Cytiva, US), 10% phosphatidylserine (Alfa Chemistry, US), and 8% cardiolipin (Merck), which were supplemented with 5% nickel-chelating 1,2-dioleoyl-sn-glycero-3-[N-(5-amino-1-carboxypentyl)iminodiacetic-acid)-succinyl] (Avanti, US) for recruitment of (His)_6_-tagged proteins to the membrane. Lipid mixtures in chloroform with 0.01% butylated hydroxytoluene (Merck) were dried under N_2_ and resuspended in a buffer containing 10 mM HEPES (pH 7.5), 135 mM KCl, and 50 mM 5(6)-carboxy-fluorescein (Cytiva). Liposomes were then passed over a PD-10 Sephadex G-25 column (Cytiva) to remove the excess dye. Liposomes were incubated with recombinant Bak and/or synthetic peptide in buffer containing 10 mM HEPES (pH 7.5) and 135 mM KCl for 0–90 min at room temperature. Fluorescence was measured by excitation at 485 nm and emission at 535 nm using an Infinite M Plex multimode microplate reader (TECAN, Switzerland).

### Cell culture

HeLa cells were cultured in Dulbecco’s Modified Eagle Medium (Corning, US) supplemented with 10% heat-inactivated fetal bovine serum (Thermo Fisher Scientific, US) and 1% penicillin/streptomycin (Thermo Fisher Scientific) at 37°C in a 5% CO_2_ incubator.

### Preparation of plasmids for the cell-based analysis and plasmid transfection

The plasmids expressing GFP-tagged human Pxt1, Pxt1(DLA), mouse Pxt1, and Flag-tagged Bcl-xL were prepared as previously described [27]. Full-length Bax (hMU011946) and Bak (hMU007094)-coding sequences were obtained from the Korea Human Gene Bank (KRIBB, Korea) and then subcloned into pcDNA3-HA.

A full-length Bim-coding sequence was synthesized from Bioneer (Korea) and subcloned into pEGFP-C2. Plasmid transfection was performed using Lipofectamine 3000 (Thermo Fisher Scientific) according to the manufacturer’s instructions.

### Immunoprecipitation

After transfection, the cells were lysed with lysis buffer containing 50 mM Tris–HCl (pH 7.5), 150 mM NaCl, 0.5% Triton X-100, 1 mM ethylenediaminetetraacetic acid, and protease inhibitor cocktail. Cell lysates were incubated with anti-GFP (Santa Cruz Biotechnology, US) or anti-HA (Santa Cruz Biotechnology) antibodies overnight at 4°C, followed by incubation with protein G agarose (Thermo Fisher Scientific) for 2 h at 4°C. Immunoprecipitated samples were eluted by incubation with sample buffer and boiled for 5 min.

### Immunoblotting

Protein samples were separated by SDS-PAGE and transferred to a nitrocellulose membrane (Thermo Fisher Scientific). The membranes were blocked with Tris-buffered saline containing 0.1% Tween-20 and 5% skim milk (BD, US) for 30 min, followed by incubation with anti-GFP, anti-HA, anti-Bcl-xL, anti-Caspase-3 (Cell Signaling Technology, US), anti-Bax (Cell Signaling Technology, Santa Cruz Biotechnology), and anti-Bak (Abcam, UK) antibodies. After incubation, the samples were incubated with goat anti-mouse IgG secondary antibody with HRP (Thermo Fisher Scientific) and goat anti-rabbit IgG secondary antibody with HRP (Enzo Life Sciences) and then analyzed using the FUSION Solo X chemiluminescence imaging system (Vilber Lourmat, France).

### Cell viability and flow cytometry analysis

HeLa cells were transfected with pEGFP-C2-mock, human Pxt1, mouse Pxt1, or human Pxt1(DLA) in the absence or presence of 20 μM z-VAD-fmk (Selleck Chemicals, US) for the indicated times. For cell viability analysis, cells were incubated with CellTiter-Glo 2.0 reagent (Promega, US) at room temperature for 15 min and then analyzed using a luminometer (SpectraMax i3; Molecular Devices, US), according to the manufacturer’s instructions. For flow cytometry analysis, cells were resuspended in annexin V binding buffer (BD Biosciences, US) and incubated with 5 μl APC annexin V (BD Biosciences) for 15 min. Annexin V–positive cells were analyzed using a BD FACSVerse flow cytometer (BD Bioscience).

### Mitochondria outer membrane potential analysis

Changes in living cells were quantified by flow cytometry using a TMRE-Mitochondrial Membrane Potential Assay Kit (Abcam). Transfected cells were incubated with 200 nM TMRE for 15 min (HeLa cells) or 30 min (*Bax*^*−/−*^ HeLa cells) in medium at 37°C. For flow cytometry analysis, incubated cells were harvested using trypsin-ethylenediaminetetraacetic acid, resuspended in phosphate-buffered saline (PBS) containing 0.2% bovine serum albumin, and analyzed using the BD FACSVerse flow cytometer.

### Confocal microscope analysis

Before cell harvesting, the cells were incubated with 500 nM MitoTracker Deep Red FM (Thermo Fisher Scientific) for 30 min to stain the mitochondria. The cells were washed with PBS and fixed by incubation with 4% paraformaldehyde (Biosesang, Korea). The fixed cells were permeabilized by incubation with PBS containing 0.2% Triton X-100 for 10 min and then incubated with PBS containing 5% bovine serum albumin (Sigma-Aldrich) for blocking. Cells were incubated with an anti-cytochrome *c* (1:100) antibody for 16 h, followed by incubation with an Alexa Fluor 546 anti-rabbit (1:100; Thermo Fisher Scientific) antibody for 1 h. The nuclei were stained by incubation with DAPI for 15 min. The samples were analyzed using an LSM 800 confocal microscope (Carl Zeiss, Germany). To promote detection of human Pxt1 and Bim, all cells were treated with 20 μM z-VAD-fmk.

### Bax knockout HeLa establishment

We used the pCGfd-gR-Cas9-GFP plasmid to generate Bax knockout HeLa cells [64]. The sgRNA primer sequences targeting exon 2 of the human *Bax* gene were designed as follows: sgRNA-oligo-F 5′-CACCGATGATCTGCTCAGAGCTGGT-3′, sgRNA-oligo-R 5′-AAACACCAGCTCTGAGCAGATCATC-3′, PAM sequence: GGG. The cells were transfected with the pCGfd-ghBax-Cas9-GFP plasmid using Lipofectamine 3000. After 24 h from transfection, limiting dilutions were performed in 96-well plates to obtain single clones. Bax deficiency was confirmed using immunoblotting and genomic DNA sequencing analysis (S2 Data).

### siRNA and transfection

siRNAs were purchased from Dharmacon (US). *Bax*^*−/−*^ HeLa cells were transfected with siNON or siBak using Lipofectamine RNAiMAX (Thermo Fisher Scientific) for 48 h followed by plasmid transfection.

### Statistical analysis

GraphPad Prism was used to perform all statistical analyses (Ver. 9.5.1; La Jolla, CA, USA). For the determination of significance, one-way analysis of variance (ANOVA) with Tukey’s honestly significant difference (HSD) was used in the confocal microscope analysis (*n =* 3), while two-way ANOVA with Tukey’s HSD was used in the TMRE analysis (*n* = 7), cell viability assays (*n* = 9 or 12), and Annexin V staining (*n* = 7). The numerical data are included in S1 Data.

## Supporting information

S1 Fig Sequence alignment. The human and mouse Pxt1 sequences were aligned. Conserved residues are shaded in navy. Four hydrophobic BH3 consensus residues are marked in green, whereas the conserved aspartate residues (Asp91 in human Pxt1) are shown in red. The box indicates the region used for crystallization. The BH3 consensus motif is also present. Ф, hydrophobic residue; Σ, small residue; Z, acidic residue; Γ, hydrophilic residue.
(TIF)

S2 Fig Immunoprecipitation assays. The indicated Pxt1, Bak, and Bax constructs were transiently expressed in HeLa cells and were subjected to co-immunoprecipitation assays. Mouse Pxt1 (**A**) and hPxt1(DLA) (**B**) are impaired in interacting with Bak or Bax. All cells were treated with 20 μM z-VAD-fmk before transfection of plasmids. Full gel figures are available in S1 Raw Images. Bak, Bcl-2 antagonist/killer; Bax, Bcl-2-associated X; DLA, alanine substitutions at Leu82 and Leu86; hPxt1, human Pxt1; mPxt1, mouse Pxt1; Pxt1, peroxisomal testis-specific 1.
(TIF)

S3 Fig Binding analyses. (**A**) SEC analysis. Recombinant Bak and the hPxt1 BH3 fragment tagged by His_10_–linked MBP were purified separately and subjected to Superdex 200 increase 10/300 GL column alone (C, samples 1 and 2) or mixed (C, samples 3). All the samples contained 1% CHAPS. The peak fractions were analyzed and visualized by SDS–PAGE and Coomassie staining. Full gel figures are available in S1 Raw Images. (**B**) ITC measurements. The indicated Pxt1 peptide (0.8 mM) was titrated into 80 μM recombinant Bak. *K*_a_, *K*_D_, and stoichiometry (N) values are shown and compared in the lower table. The numerical data are included in S1 Data. Bak, Bcl-2 antagonist/killer; CHAPS, 3-[(3-cholamidopropyl)dimethylammonio]-1-propanesulfonate; hPxt1, human Pxt1; ITC, isothermal titration calorimetry; *K*_a,_ association constant; *K*_D_, dissociation constant, MBP, maltose binding protein; PAGE, polyacrylamide gel electrophoresis; Pxt1, peroxisomal testis-specific 1; S, size marker; SDS, sodium dodecyl sulfate; SEC, size-exclusion chromatography.
(TIF)

S4 Fig Structural comparison with previously determined structures. (**A**) Pxt1-bound Bcl-xL and Bak structures are superimposed. Five BH3 consensus residues are shown as sticks with labels. (**B**) Bak molecules in the Pxt1-superposed apo form (left; PDB code 2IMT) or in the Pxt1-complexed form (right) are shown in the electrostatic surface representation together with the Pxt1 (green) fragment. Bak, Bcl-2 antagonist/killer; Pxt1, peroxisomal testis-specific 1.
(TIF)

S5 Fig Representative carboxyfluorescein release in the liposome assay. The graphs represent carboxyfluorescein release at the indicated moment in the liposome assay show in Fig 4C. DLA, human Pxt1(76–101) peptide containing alanine substitutions at Leu82 and Leu86. Experiments were performed in independent triplicate, and the numerical data are included in S1 Data.
(TIF)

S6 Fig Analysis of protein levels. Full gel figures are available in S1 Raw Images. (**A**, **B**) Immunoblotting analysis for detection of the indicated Pxt1 (**A**) or Bim (**B**) constructs transiently expressed in HeLa cells. Asterisks, nonspecific bands. (**C**) Gene knockout/knockdown of Bax/Bak. (Left) Four Bax-deficient HeLa cell lines were prepared using CRISPR technology. Among them, the *Bax*^−/−^ cell line #4 (marked in red) was used in this study, whose establishment is shown in S2 Data. (Right) Protein levels of Bak in *Bax*^−/−^ HeLa cells were analyzed upon siNON or siBak treatment. In this study, 10 nM siNON and siBak (marked in red) were used. (**D**) Immunoblotting analysis for detection of hPxt1 transiently expressed in *Bax*^−/−^ HeLa cells. Bak, Bcl-2 antagonist/killer; Bax, Bcl-2-associated X; hPxt1, human Pxt1; mPxt1, mouse Pxt1; Pxt1, peroxisomal testis-specific 1.
(TIF)

S7 Fig hPxt1 and Bim are similarly effective as apoptogenic factors. (**A**) CellTiter-Glo assay was used to measure viability of HeLa cells transiently expressing GFP-tagged full-length human Pxt1 or Bim. z-VAD-fmk was used to inhibit apoptosis. Experiments were performed in 12 replicates. ns, nonsignificant; ****, *P* < 0.0001 in the two-way ANOVA followed by Tukey’s HSD. The numerical data are included in S1 Data. (**B**) Cleavage of Pro-caspase 3 was analyzed by immunoblotting after transfection of Pxt1 or Bim constructs. *, nonspecific band. Full gel figures are available in S1 Raw Images. ANOVA, analysis of variance; GFP, green fluorescent protein; hPxt1, human Pxt1; HSD, honestly significant difference; Pxt1, peroxisomal testis-specific 1.
(TIF)

S8 Fig Introduction of L82A·L86A mutation impairs proapoptotic activity of Pxt1. Wild-type human Pxt1, Pxt1(DLA), or Bim was transiently expressed in HeLa cells for flow cytometry analysis. The ratios of annexin V–positive and negative cells are shown in red and blue, respectively (left), and are represented as graphs (right). Experiments were performed in 7 replicates. ns, nonsignificant; ****, *P* < 0.0001 in the two-way ANOVA followed by Tukey’s HSD. The numerical data are included in S1 Data. The gating strategies of flow cytometry are included in S3C Data. ANOVA, analysis of variance; APC, allophycocyanin; DLA, alanine substitutions at Leu82 and Leu86; FSC-A, forward scatter area; HSD, honestly significant difference; Pxt1, peroxisomal testis-specific 1.
(TIF)

S1 Data Numerical data underlying Figs 1D, 4C, 5A, 5B, 6A, 6B, 7A, 7B, 8A, 8B, S3B, S5, S7A, and S8. (XLSX)

S2 Data Establishment of *Bax*^−/−^ cell lines underlying S6C Fig. (PDF)

S3 Data Gating strategies of the flow cytometry analyses. (**A**) MOMP analysis using TMRE underlying Figs 5A and 7A. (**B**) GFP expression and cell death analysis underlying Fig 6D–6F. (**C**) Cell death analysis underlying Figs 8B and S8.
(PDF)

S1 Raw Images Full gel figures underlying Figs 1A–C, 4A–B, 6C, S2A, S2B, S3A, S6A–S6D, and S7B. (PDF)

S1 Table Data collection and structure refinement statistics. (DOCX)

## Abbreviations

ANOVA: analysis of variance
APC: allophycocyanin
Bak: Bcl-2 antagonist/killer
Bax: Bcl-2-associated X
Bcl-2: B cell lymphoma-2
CHAPS: 3-[(3-cholamidopropyl)dimethylammonio]-1-propanesulfonate
DAPI: 4′,6-diamidino-2-phenylindole
GFP: green fluorescent protein
HSD: honestly significant difference
ITC: isothermal titration calorimetry
*K*_D_: dissociation constant, MBP, maltose binding protein
MOMP: mitochondrial outer membrane permeabilization
NTA: Ni–nitrilotriacetic acid
PAGE: polyacrylamide gel electrophoresis
PBS: phosphate-buffered saline
Pxt1: peroxisomal testis-specific 1
SDS: sodium dodecyl sulfate
SEC: size-exclusion chromatography
TMRE: tetramethylrhodamine ethyl ester
ΔΨm: mitochondrial membrane potential
