# Supplementary figures and images for: Structural basis for proapoptotic activation of Bak by the noncanonical BH3-only protein Pxt1
Source: PLoS Biol. 2023 Jun 14;21(6):e3002156. doi: 10.1371/journal.pbio.3002156 (PMC10298792; doi:10.1371/journal.pbio.3002156)

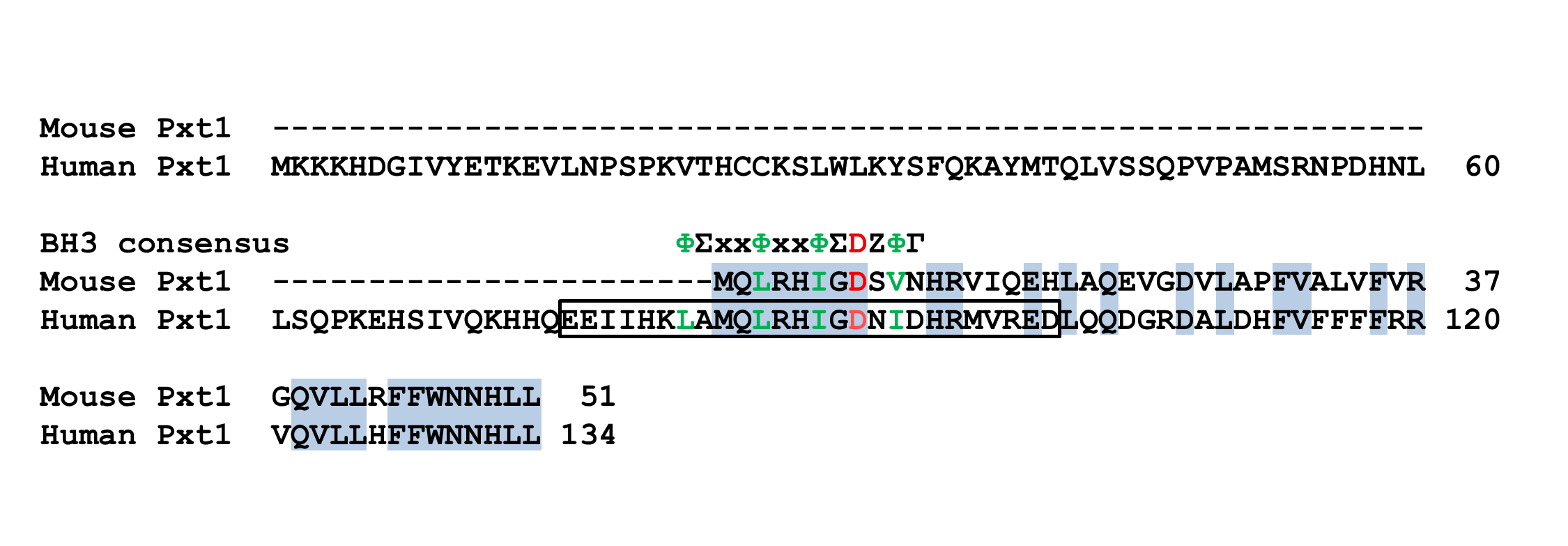

Supplement: S1 Fig — The human and mouse Pxt1 sequences were aligned. Conserved residues are shaded in navy. Four hydrophobic BH3 consensus residues are marked in green, whereas the conserved aspartate residues (Asp91 in human Pxt1) are shown in red. The box indicates the region used for crystallization. The BH3 consensus motif is also present. Ф, hydrophobic residue; Σ, small residue; Z, acidic residue; Γ, hydrophilic residue. (TIF) [file pbio.3002156.s001.tif]

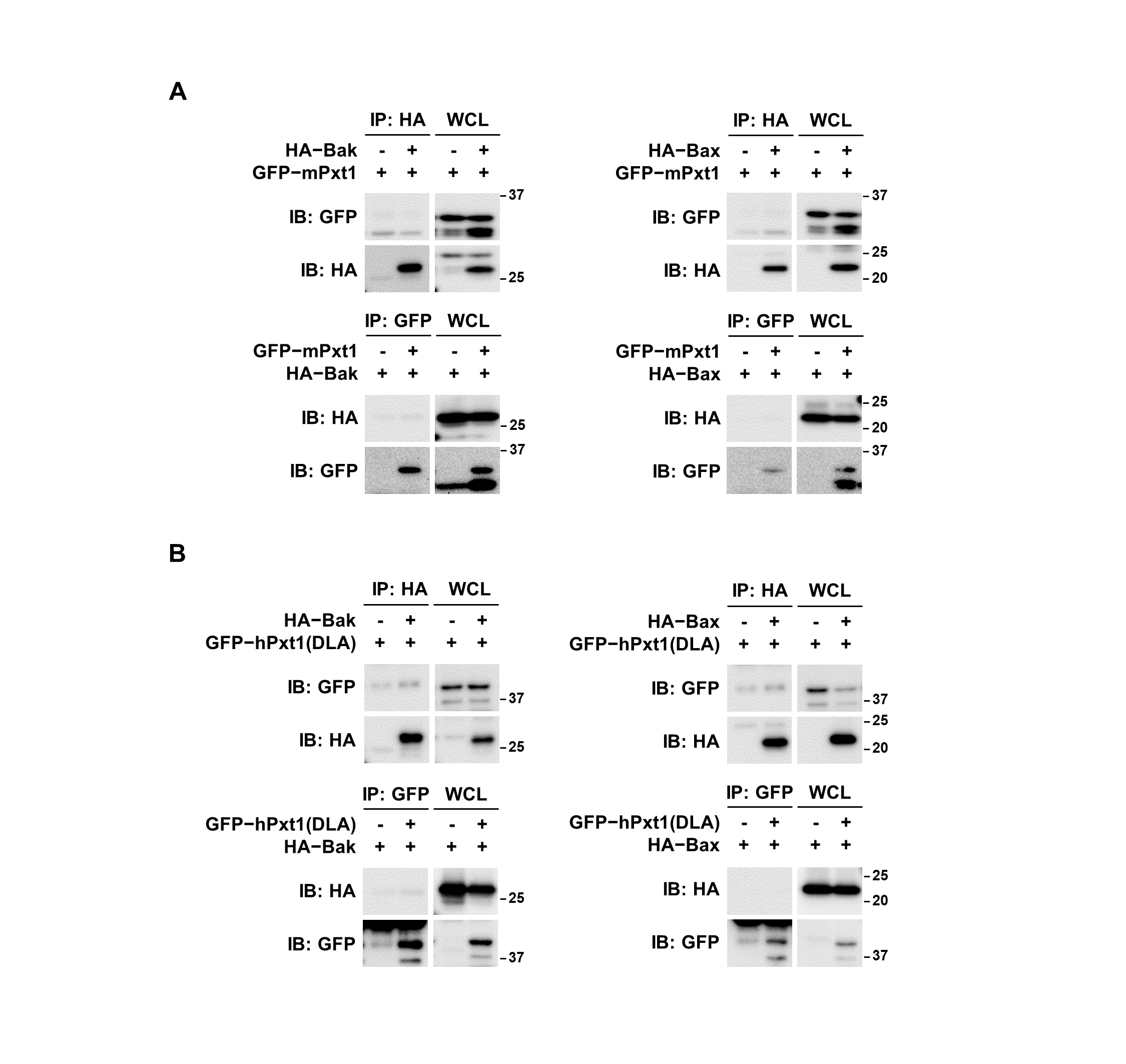

Supplement: S2 Fig — The indicated Pxt1, Bak, and Bax constructs were transiently expressed in HeLa cells and were subjected to co-immunoprecipitation assays. Mouse Pxt1 (A) and hPxt1(DLA) (B) are impaired in interacting with Bak or Bax. All cells were treated with 20 μM z-VAD-fmk before transfection of plasmids. Full gel figures are available in S1 Raw Images. Bak, Bcl-2 antagonist/killer; Bax, Bcl-2-associated X; DLA, alanine substitutions at Leu82 and Leu86; hPxt1, human Pxt1; mPxt1, mouse Pxt1; Pxt1, peroxisomal testis-specific 1. (TIF) [file pbio.3002156.s002.tif]

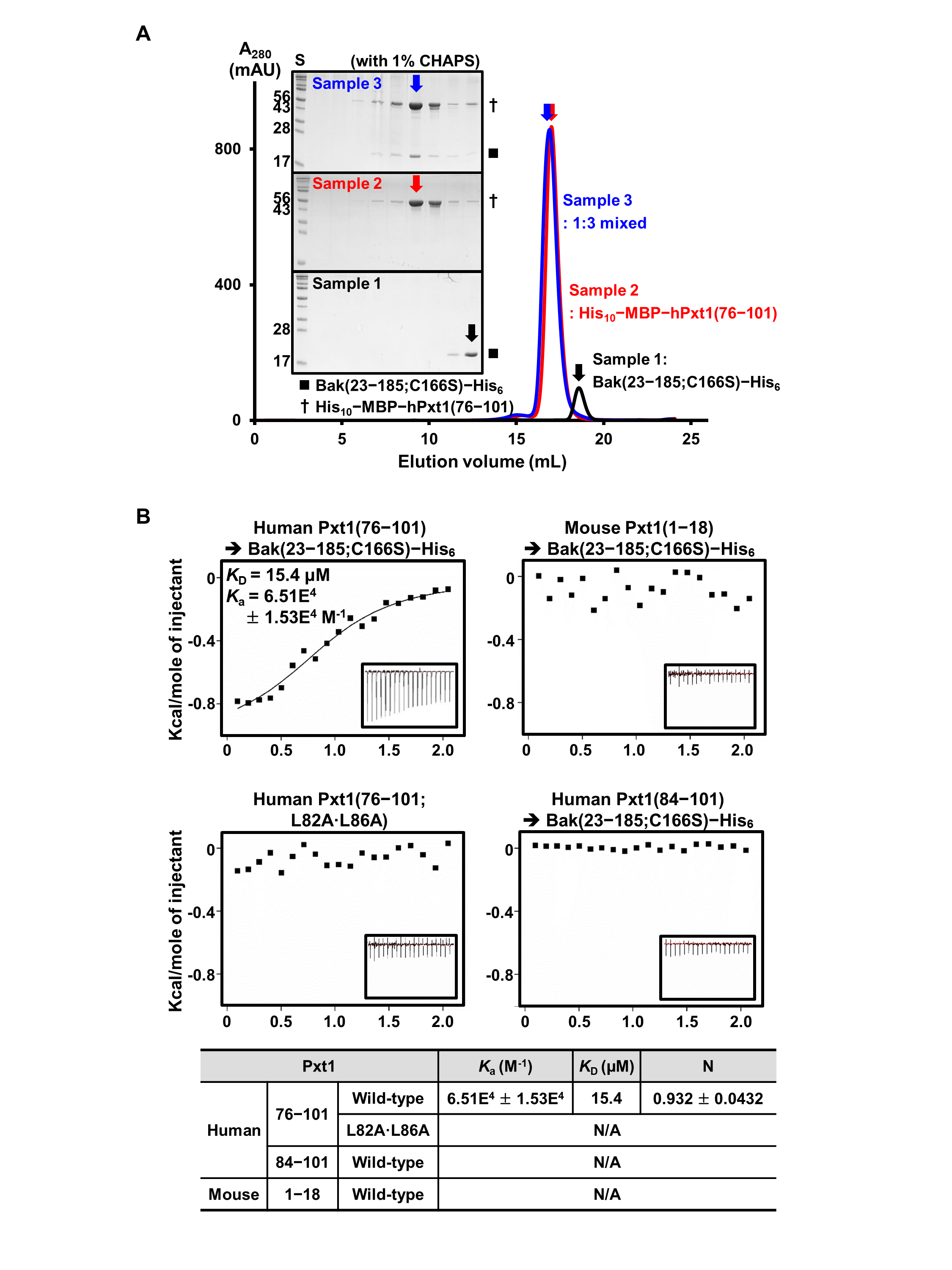

Supplement: S3 Fig — (A) SEC analysis. Recombinant Bak and the hPxt1 BH3 fragment tagged by His10–linked MBP were purified separately and subjected to Superdex 200 increase 10/300 GL column alone (C, samples 1 and 2) or mixed (C, samples 3). All the samples contained 1% CHAPS. The peak fractions were analyzed and visualized by SDS–PAGE and Coomassie staining. Full gel figures are available in S1 Raw Images. (B) ITC measurements. The indicated Pxt1 peptide (0.8 mM) was titrated into 80 μM recombinant Bak. Ka, KD, and stoichiometry (N) values are shown and compared in the lower table. The numerical data are included in S1 Data. Bak, Bcl-2 antagonist/killer; CHAPS, 3-[(3-cholamidopropyl)dimethylammonio]-1-propanesulfonate; hPxt1, human Pxt1; ITC, isothermal titration calorimetry; Ka, association constant; KD, dissociation constant, MBP, maltose binding protein; PAGE, polyacrylamide gel electrophoresis; Pxt1, peroxisomal testis-specific 1; S, size marker; SDS, sodium dodecyl sulfate; SEC, size-exclusion chromatography. (TIF) [file pbio.3002156.s003.tif]

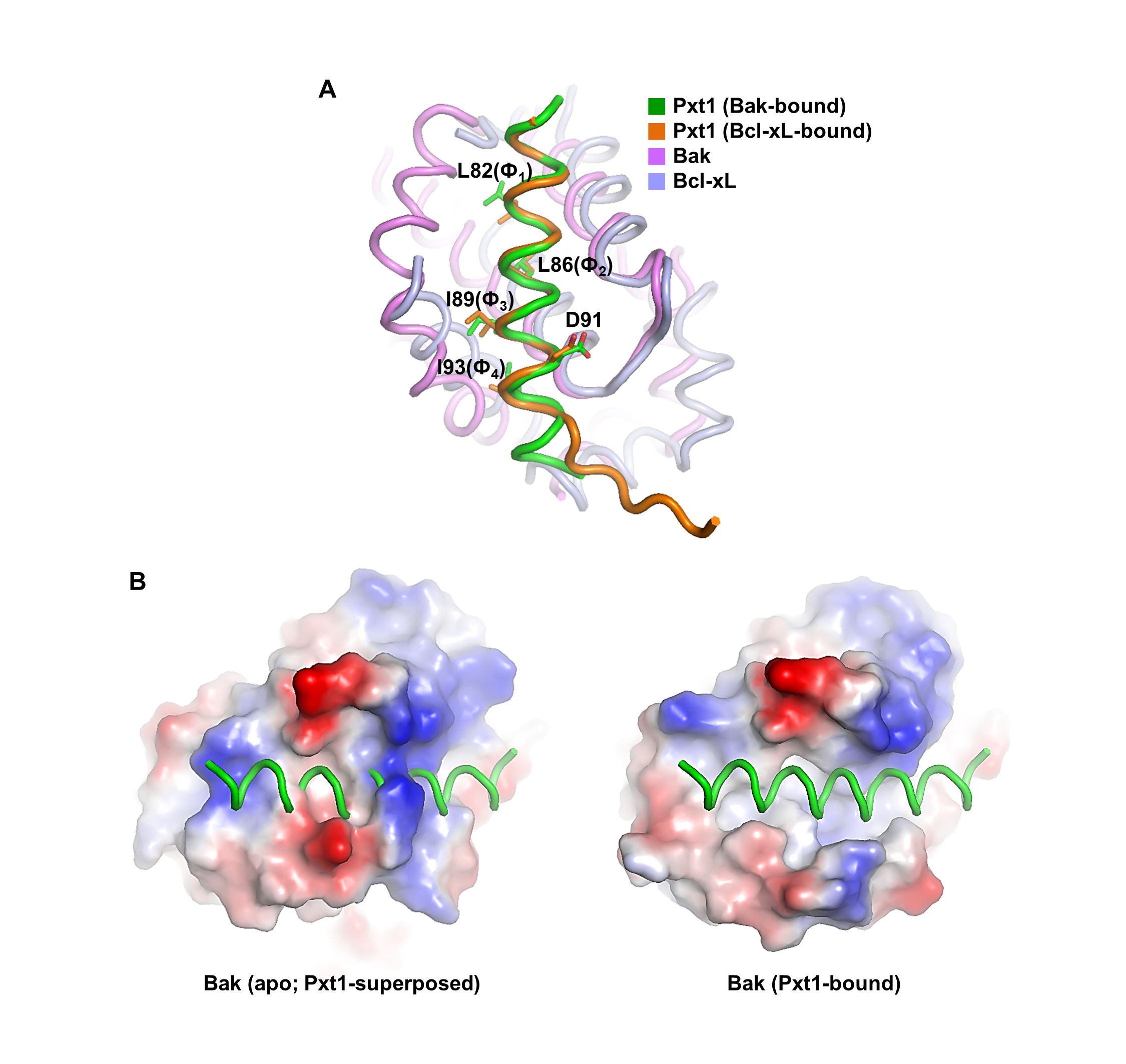

Supplement: S4 Fig — (A) Pxt1-bound Bcl-xL and Bak structures are superimposed. Five BH3 consensus residues are shown as sticks with labels. (B) Bak molecules in the Pxt1-superposed apo form (left; PDB code 2IMT) or in the Pxt1-complexed form (right) are shown in the electrostatic surface representation together with the Pxt1 (green) fragment. Bak, Bcl-2 antagonist/killer; Pxt1, peroxisomal testis-specific 1. (TIF) [file pbio.3002156.s004.tif]

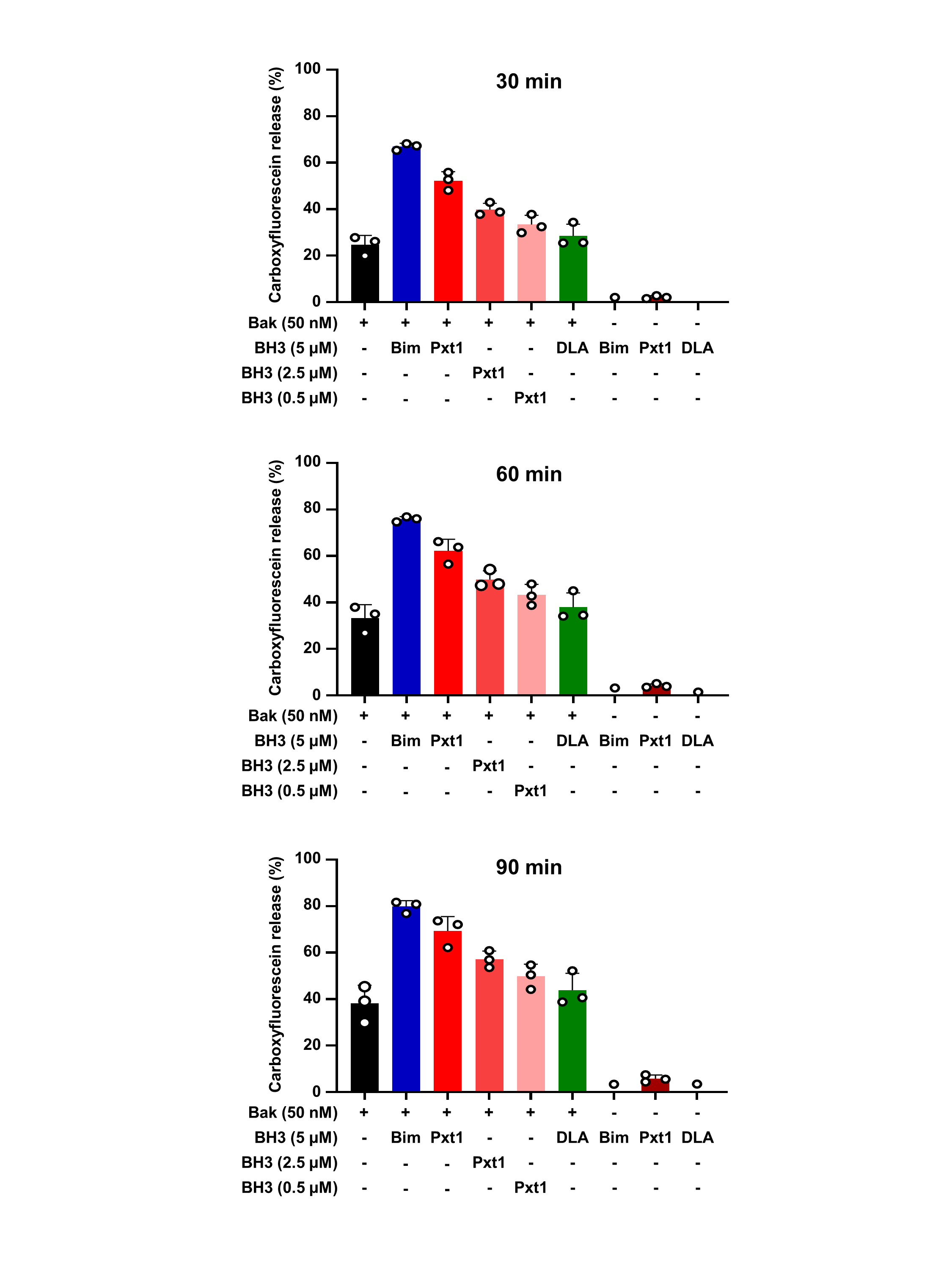

Supplement: S5 Fig — The graphs represent carboxyfluorescein release at the indicated moment in the liposome assay show in Fig 4C. DLA, human Pxt1(76–101) peptide containing alanine substitutions at Leu82 and Leu86. Experiments were performed in independent triplicate, and the numerical data are included in S1 Data. (TIF) [file pbio.3002156.s005.tif]

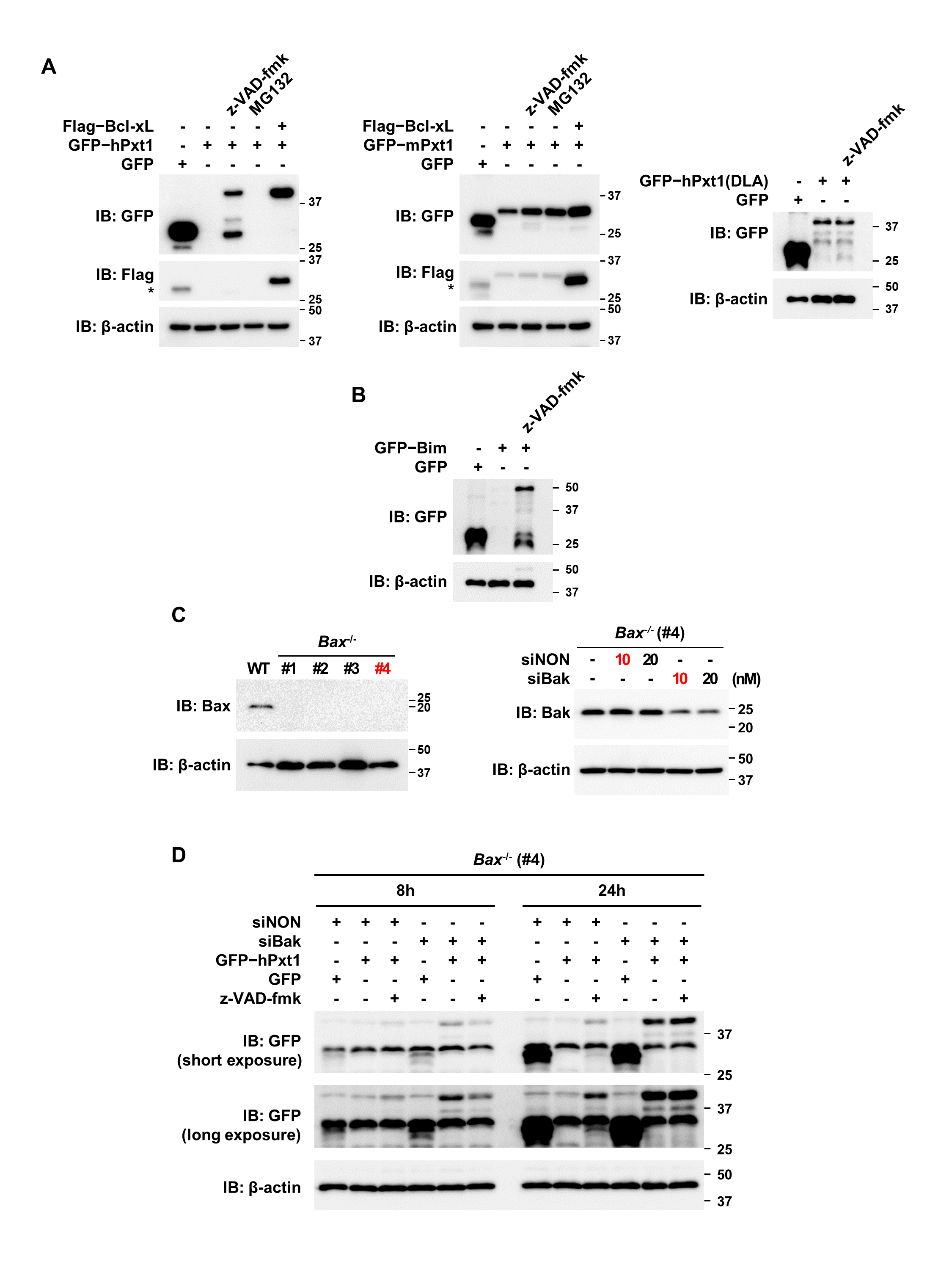

Supplement: S6 Fig — Full gel figures are available in S1 Raw Images. (A, B) Immunoblotting analysis for detection of the indicated Pxt1 (A) or Bim (B) constructs transiently expressed in HeLa cells. Asterisks, nonspecific bands. (C) Gene knockout/knockdown of Bax/Bak. (Left) Four Bax-deficient HeLa cell lines were prepared using CRISPR technology. Among them, the Bax−/− cell line #4 (marked in red) was used in this study, whose establishment is shown in S2 Data. (Right) Protein levels of Bak in Bax−/− HeLa cells were analyzed upon siNON or siBak treatment. In this study, 10 nM siNON and siBak (marked in red) were used. (D) Immunoblotting analysis for detection of hPxt1 transiently expressed in Bax−/− HeLa cells. Bak, Bcl-2 antagonist/killer; Bax, Bcl-2-associated X; hPxt1, human Pxt1; mPxt1, mouse Pxt1; Pxt1, peroxisomal testis-specific 1. (TIF) [file pbio.3002156.s006.tif]

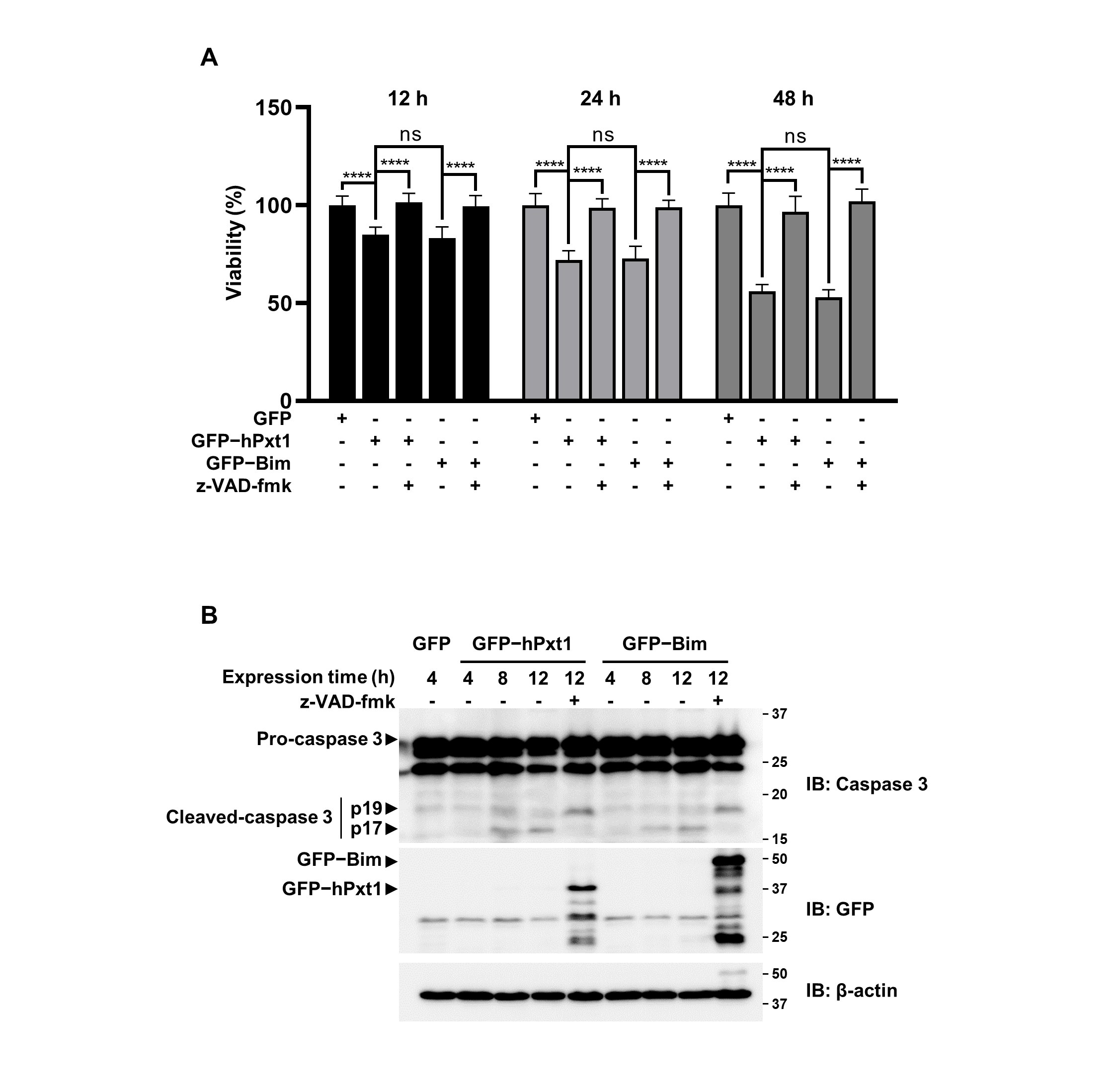

Supplement: S7 Fig — (A) CellTiter-Glo assay was used to measure viability of HeLa cells transiently expressing GFP-tagged full-length human Pxt1 or Bim. z-VAD-fmk was used to inhibit apoptosis. Experiments were performed in 12 replicates. ns, nonsignificant; ****, P < 0.0001 in the two-way ANOVA followed by Tukey’s HSD. The numerical data are included in S1 Data. (B) Cleavage of Pro-caspase 3 was analyzed by immunoblotting after transfection of Pxt1 or Bim constructs. *, nonspecific band. Full gel figures are available in S1 Raw Images. ANOVA, analysis of variance; GFP, green fluorescent protein; hPxt1, human Pxt1; HSD, honestly significant difference; Pxt1, peroxisomal testis-specific 1. (TIF) [file pbio.3002156.s007.tif]

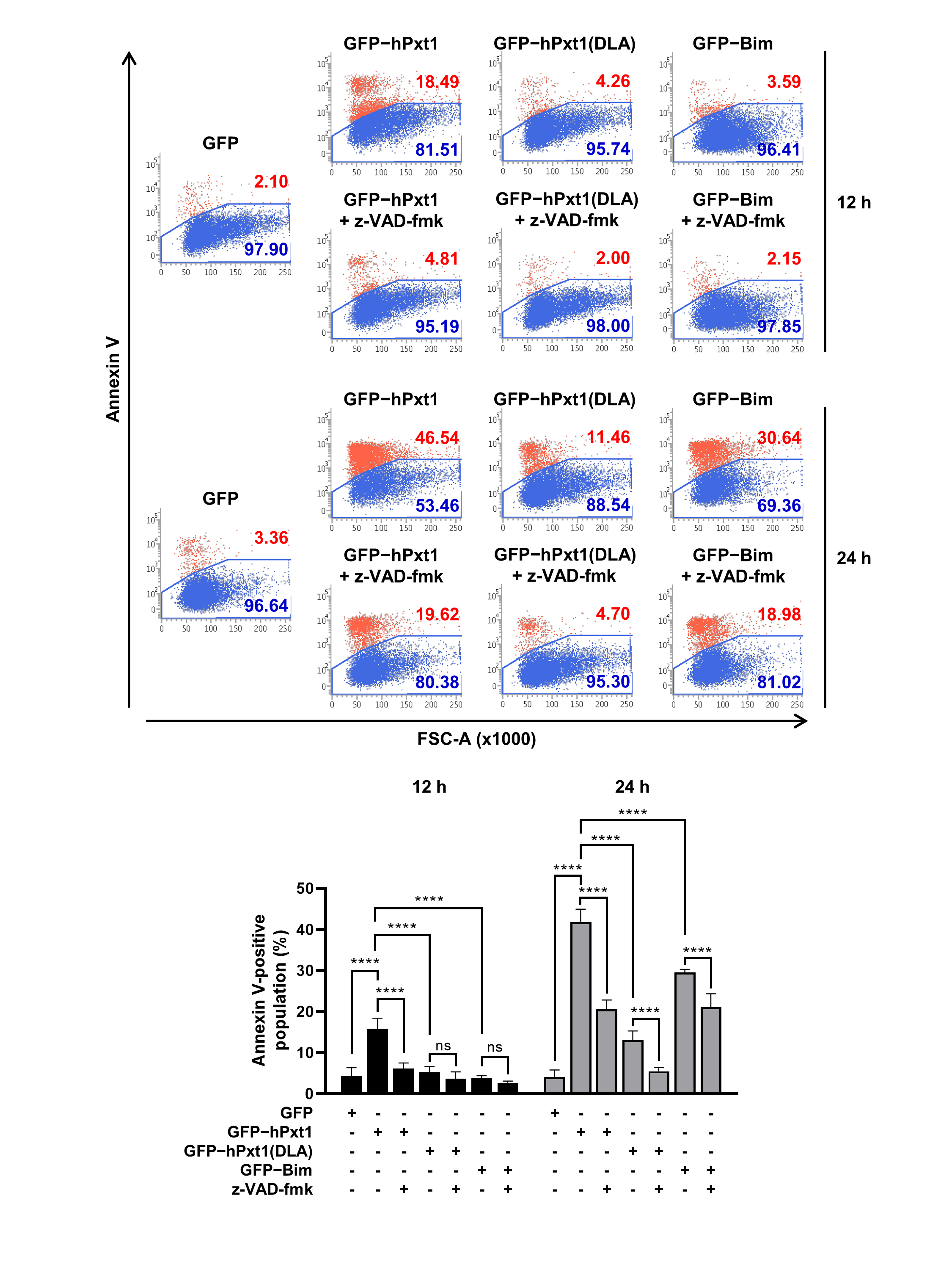

Supplement: S8 Fig — Wild-type human Pxt1, Pxt1(DLA), or Bim was transiently expressed in HeLa cells for flow cytometry analysis. The ratios of annexin V–positive and negative cells are shown in red and blue, respectively (left), and are represented as graphs (right). Experiments were performed in 7 replicates. ns, nonsignificant; ****, P < 0.0001 in the two-way ANOVA followed by Tukey’s HSD. The numerical data are included in S1 Data. The gating strategies of flow cytometry are included in S3C Data. ANOVA, analysis of variance; APC, allophycocyanin; DLA, alanine substitutions at Leu82 and Leu86; FSC-A, forward scatter area; HSD, honestly significant difference; Pxt1, peroxisomal testis-specific 1. (TIF) [file pbio.3002156.s008.tif]

**A**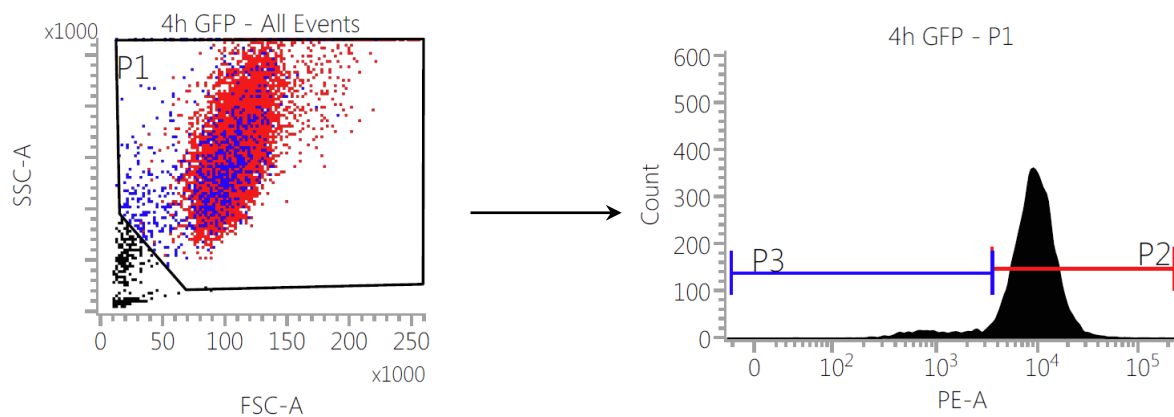**B**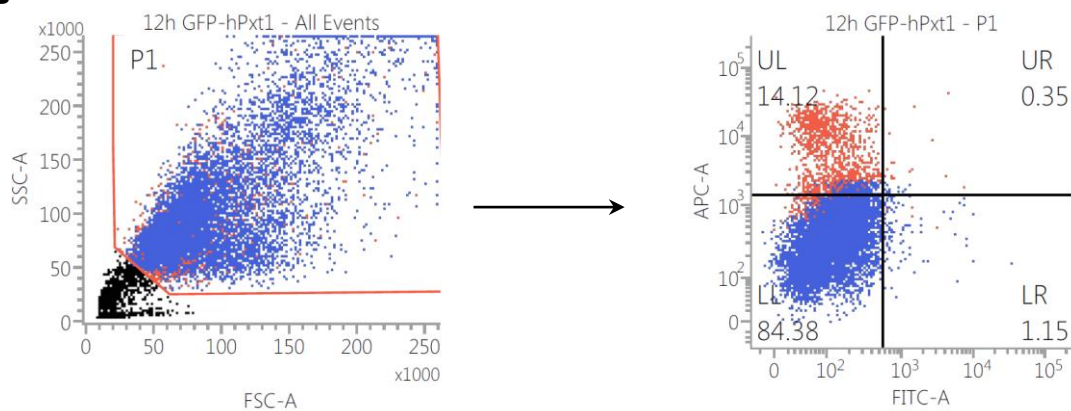**C**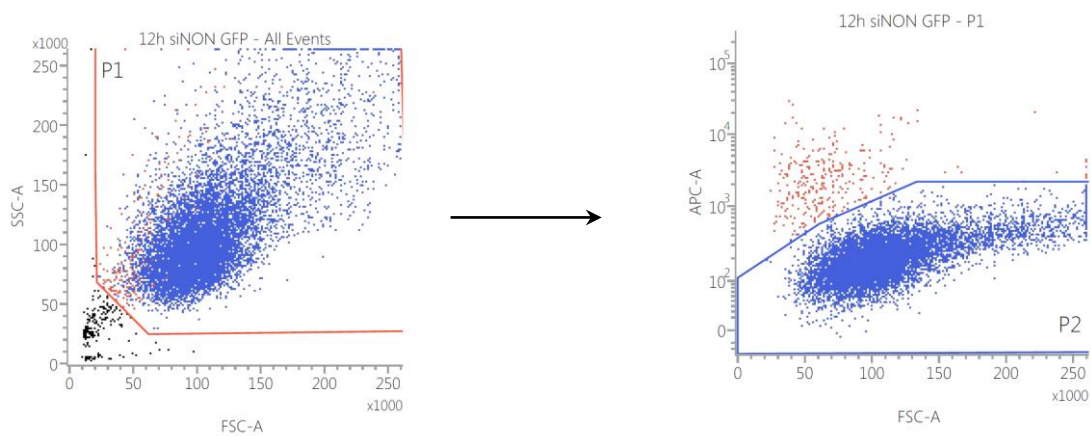

Supplement: S3 Data — (A) MOMP analysis using TMRE underlying Figs 5A and 7A. (B) GFP expression and cell death analysis underlying Fig 6D–6F. (C) Cell death analysis underlying Figs 8B and S8. (PDF) [file pbio.3002156.s011.pdf]
