# Supplementary material for: Structural basis for proapoptotic activation of Bak by the noncanonical BH3-only protein Pxt1
Source: PLoS Biol. 2023 Jun 14;21(6):e3002156. doi: 10.1371/journal.pbio.3002156 (PMC10298792; doi:10.1371/journal.pbio.3002156)
Supplement: S2 Data — (PDF) [file pbio.3002156.s010.pdf]

# Establishment of *Bax*$^{-/-}$ HeLa cells

**Insertion of adenine between A43 and G44**

## Wild-type

```
 M   D   G   S   G   E   Q   P   R   G   G   G   P   T   S   S   E   Q   I   M   K   T   G   A   L   L   L   Q   G   F   I   Q   D   R   A   G   R   M   G
ATG GAC GGG TCC GGG GAG CAG CCC AGA GGC GGG GGG CCC ACC AGC TCT GAG CAG ATC ATG AAG ACA GGG GCC CTT TTG CTT CAG GGT TTC ATC CAG GAT CGA GCA GGG CGA ATG GGG
 G   E   A   P   E   L   A   L   D   P   V   P   Q   D   A   S   T   K   K   L   S   E   C   L   K   R   I   G   D   E   L   D   S   N   M   E   L   Q   R
GGG GAG GCA CCC GAG CTG GCC CTG GAC CCG GTG CCT CAG GAT GCG TCC ACC AAG AAG CTG AGC GAG TGT CTC AAG CGC ATC GGG GAC GAA CTG GAC AGT AAC ATG GAG CTG CAG AGG
 M   I   A   A   V   D   T   D   S   P   R   E   V   F   F   R   V   A   A   D   M   F   S   D   G   N   F   N   W   G   R   V   V   A   L   F   Y   F   A
ATG ATT GCC GCC GTG GAC ACA GAC TCC CCC CGA GAG GTC TTT TTC CGA GTG GCA GCT GAC ATG TTT TCT GAC GGC AAC TTC AAC TGG GGC CGG GTT GTC GCC CTT TTC TAC TTT GCC
 S   K   L   V   L   K   A   L   C   T   K   V   P   E   L   I   R   T   I   M   G   W   T   L   D   F   L   R   E   R   L   L   G   W   I   Q   D   Q   G
AGC AAA CTG GTG CTC AAG GCC CTG TGC ACC AAG GTG CCG GAA CTG ATC AGA ACC ATC ATG GGC TGG ACA TTG GAC TTC CTC CGG GAG CGG CTG TTG GGC TGG ATC CAA GAC CAG GGT
 G   W   D   G   L   L   S   Y   F   G   T   P   T   W   Q   T   V   T   I   F   V   A   G   V   L   T   A   S   L   T   I   W   K   K   M   G   *
GGT TGG GAC GGC CTC CTC TCC TAC TTT GGG ACG CCC ACG TGG CAG ACC GTG ACC ATC TTT GTG GCG GGA GTG CTC ACC GCC TCA CTC ACC ATC TGG AAG AAG ATG GGC TGA
```

## *Bax*$^{-/-}$

```
 M   D   G   S   G   E   Q   P   R   G   G   G   P   T   K   L   *   A   D   H   E   D   R   G   P   F   A   S   G   F   H   P   G   S   S   R   A   N   G
ATG GAC GGG TCC GGG GAG CAG CCC AGA GGC GGG GGG CCC ACC AAG CTC TGA GCA GAT CAT GAA GAC AGG GGC CCT TTT GCT TCA GGG TTT CAT CCA GGA TCG AGC AGG GCG AAT GGG
 G   G   G   T   R   A   G   P   G   P   G   A   S   G   C   V   H   Q   E   A   E   R   V   S   Q   A   H   R   G   R   T   G   Q   *   H   G   A   A   E
GGG GGA GGC ACC CGA GCT GGC CCT GGA CCC GGT GCC TCA GGA TGC GTC CAC CAA GAA GCT GAG CGA GTG TCT CAA GCG CAT CGG GGA CGA ACT GGA CAG TAA CAT GGA GCT GCA GAG
 D   D   C   R   R   G   H   R   L   P   P   R   G   L   F   P   S   G   S   *   H   V   F   *   R   Q   L   Q   L   G   P   G   C   R   P   F   L   L   C
GAT GAT TGC CGC CGT GGA CAC AGA CTC CCC CCG AGA GGT CTT TTT CCG AGT GGC AGC TGA CAT GTT TTC TGA CGG CAA CTT CAA CTG GGG CCG GGT TGT CGC CCT TTT CTA CTT TGC
 Q   Q   T   G   A   Q   G   P   V   H   Q   G   A   G   T   D   Q   N   H   H   G   L   D   I   G   L   P   P   G   A   A   V   G   L   D   P   R   P   G
CAG CAA ACT GGT GCT CAA GGC CCT GTG CAC CAA GGT GCC GGA ACT GAT CAG AAC CAT CAT GGG CTG GAC ATT GGA CTT CCT CCG GGA GCG GCT GTT GGG CTG GAT CCA AGA CCA GGG
 W   L   G   R   P   P   L   L   L   W   D   A   H   V   A   D   R   D   H   L   C   G   G   S   A   H   R   L   T   H   H   L   E   E   D   G   L
TGG TTG GGA CGG CCT CCT CTC CTA CTT TGG GAC GCC CAC GTG GCA GAC CGT GAC CAT CTT TGT GGC GGG AGT GCT CAC CGC CTC ACT CAC CAT CTG GAA GAA GAT GGG CTG A
```

**Alignment of the Bax sequences between wild-type and *Bax*-knocked out (*Bax*$^{-/-}$) clones.**

**Yellow background represents the PAM sequence.**

**Green background indicates the single guide RNA sequence targeting Bax.**

**Red background shows the position of single base pair inserted for genetic ablation of Bax.**

**Red letters indicate stop codons induced by base pair insertion in the *Bax*$^{-/-}$ clone.**

**Red circles represents all the stop codons in the two clones.**
