# Supplementary material for: Structural basis for proapoptotic activation of Bak by the noncanonical BH3-only protein Pxt1
Source: PLoS Biol. 2023 Jun 14;21(6):e3002156. doi: 10.1371/journal.pbio.3002156 (PMC10298792; doi:10.1371/journal.pbio.3002156)

Fig. 1A – left, top

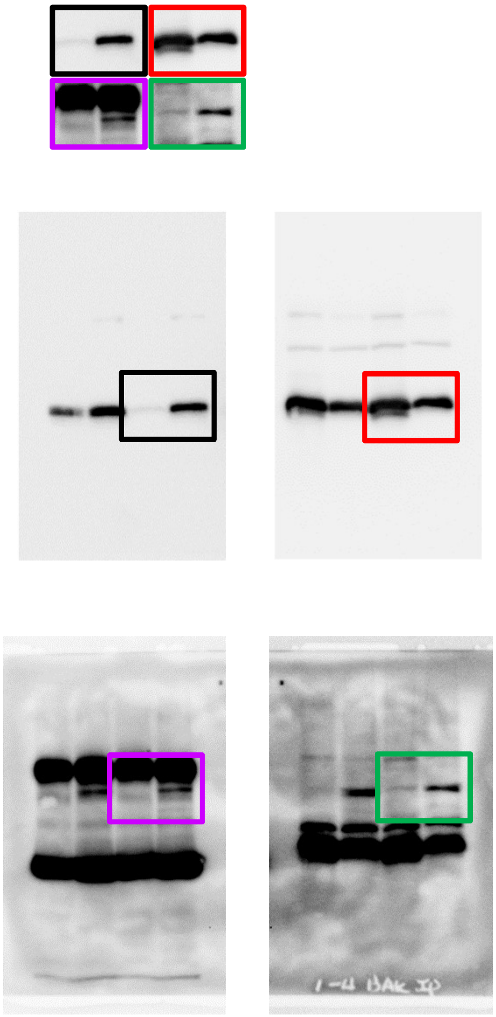

Fig. 1A – right, top

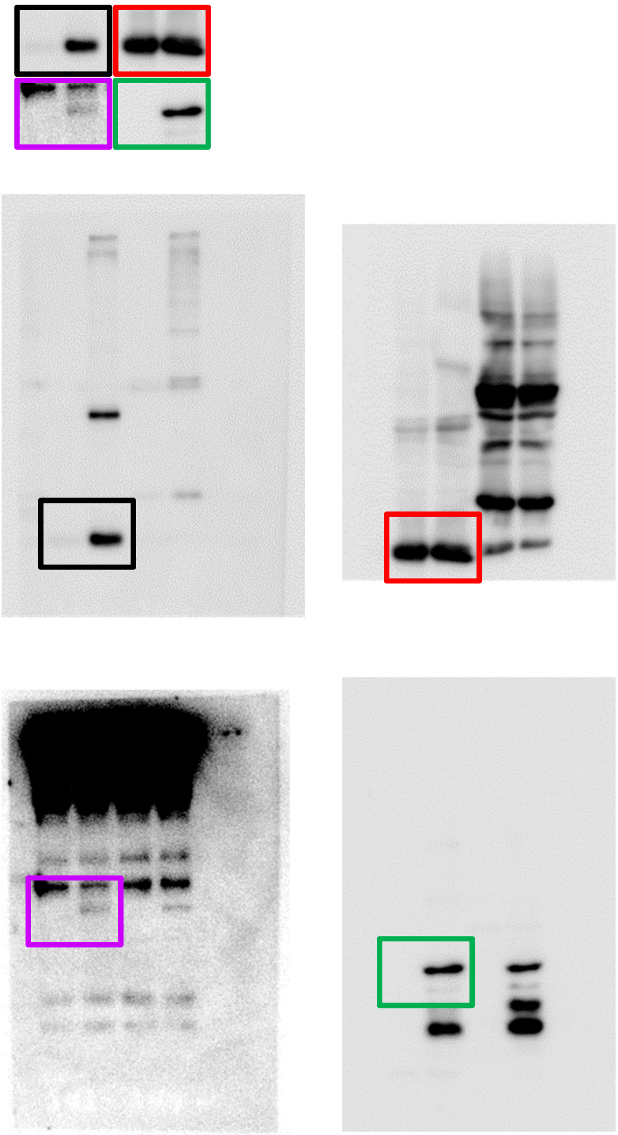

Fig. 1A – left, bottom

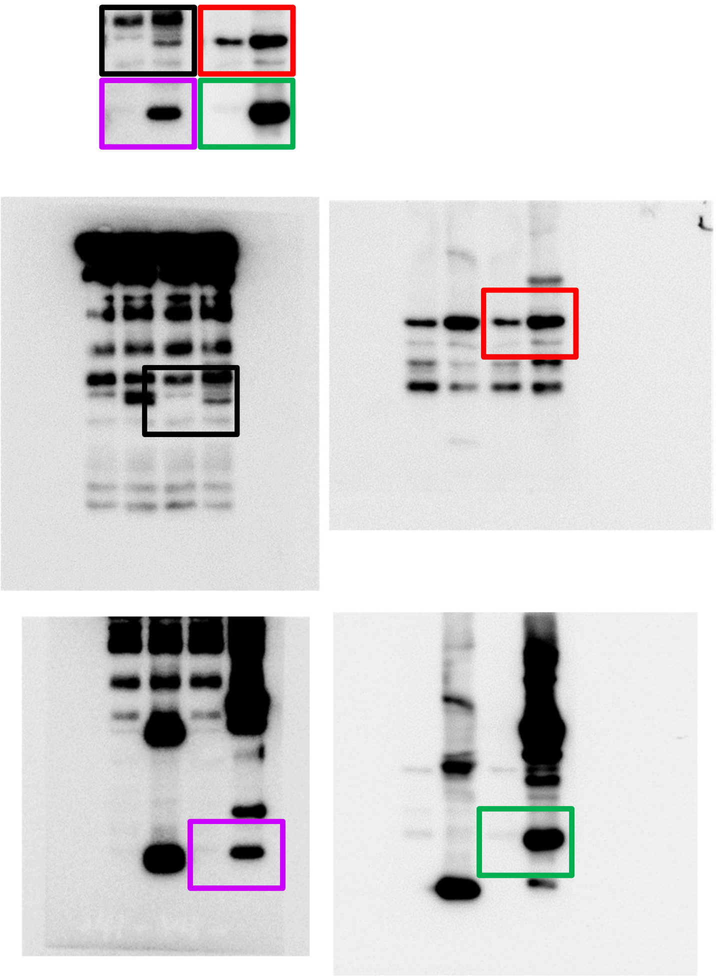

Fig. 1A – right, bottom

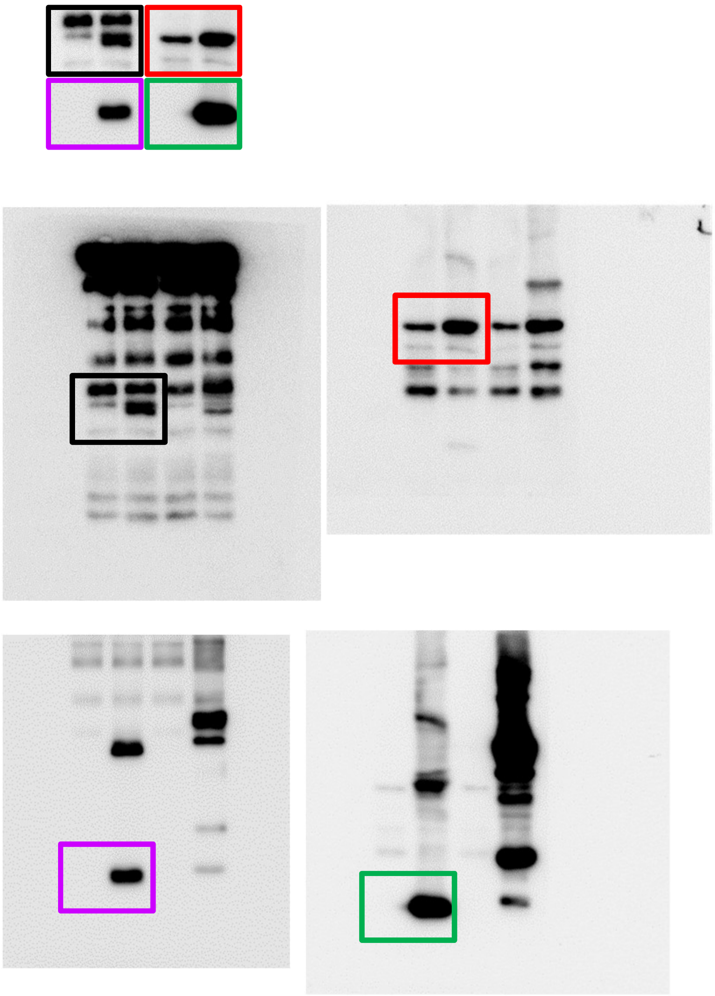

Fig. 1B

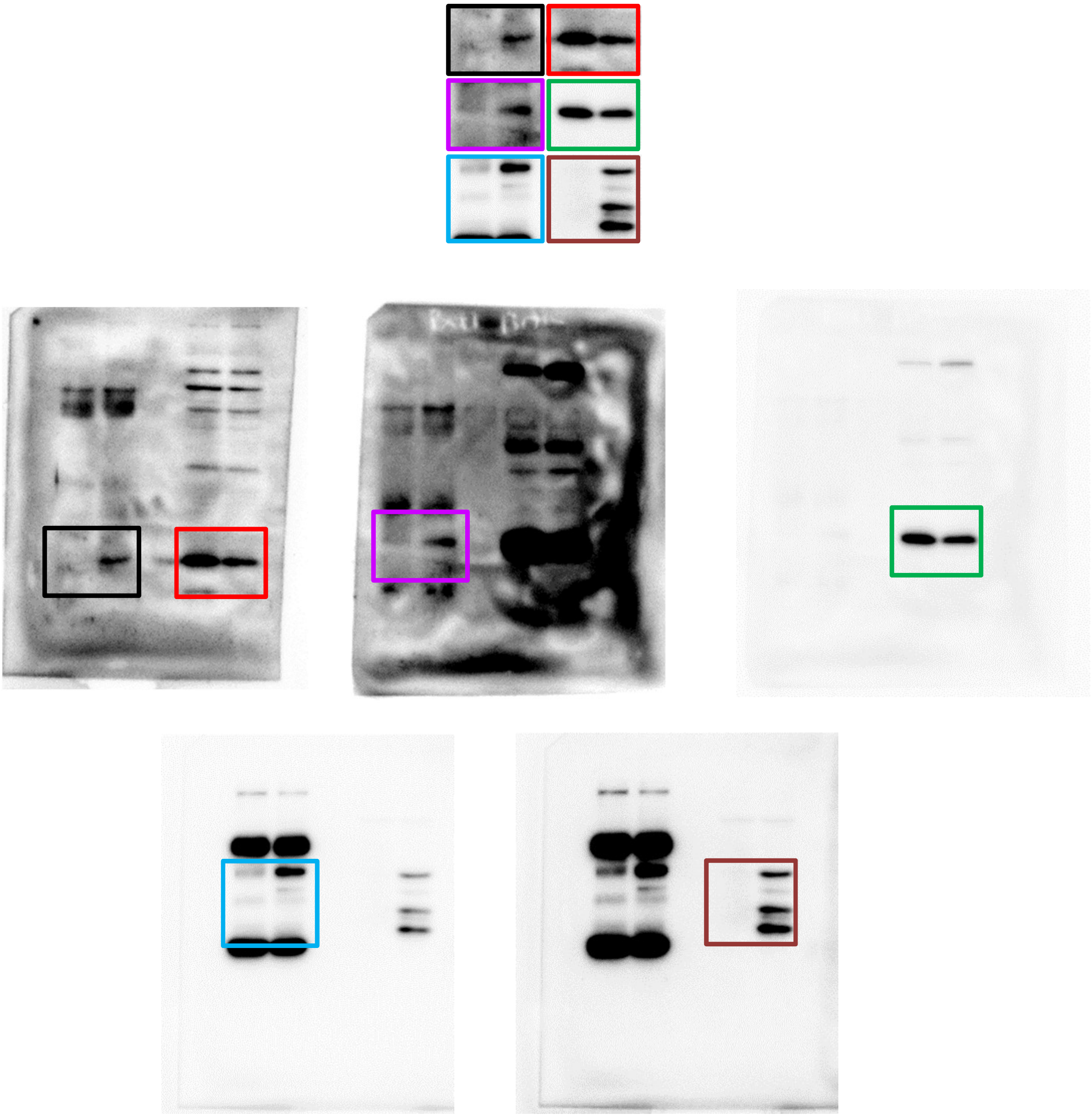

Fig. 1C – left

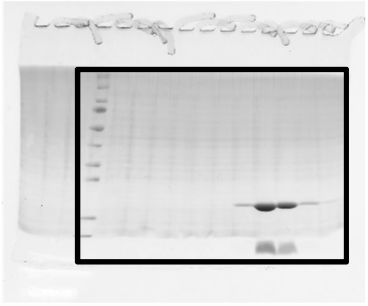

Fig. 1C– right

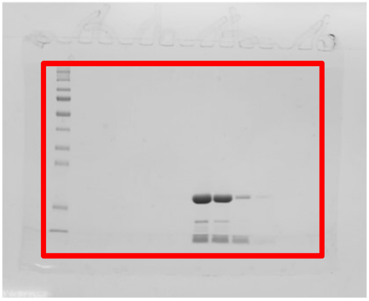

Fig. 4A

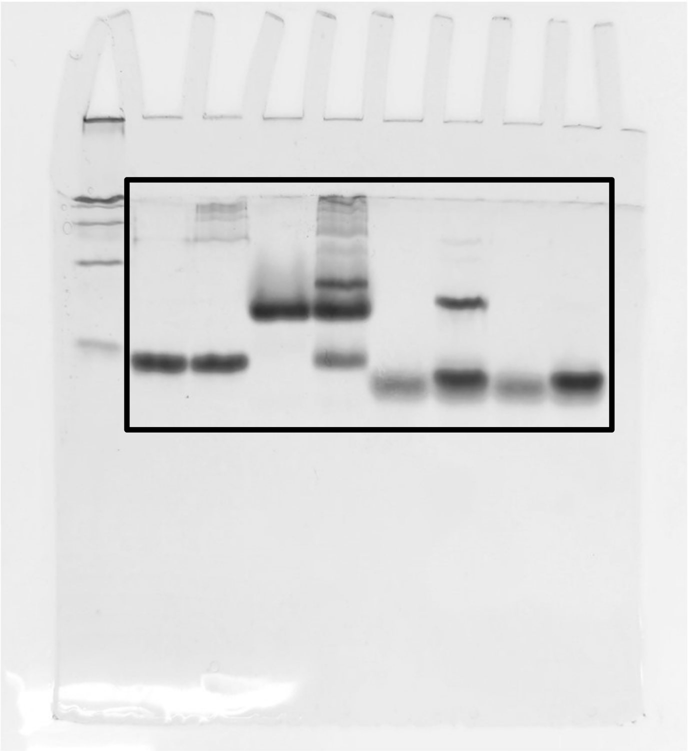

Fig. 4B

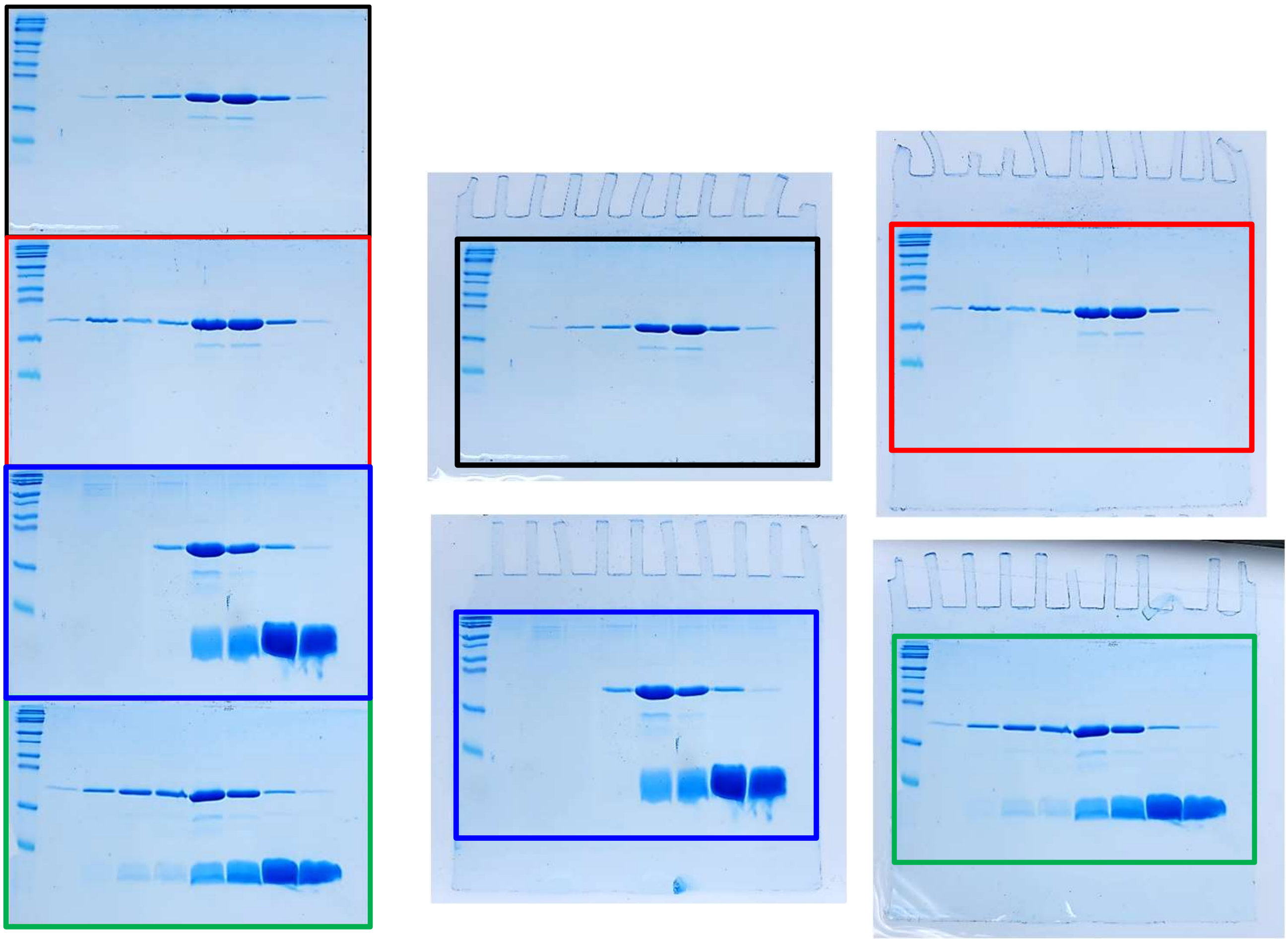

Fig. 6C

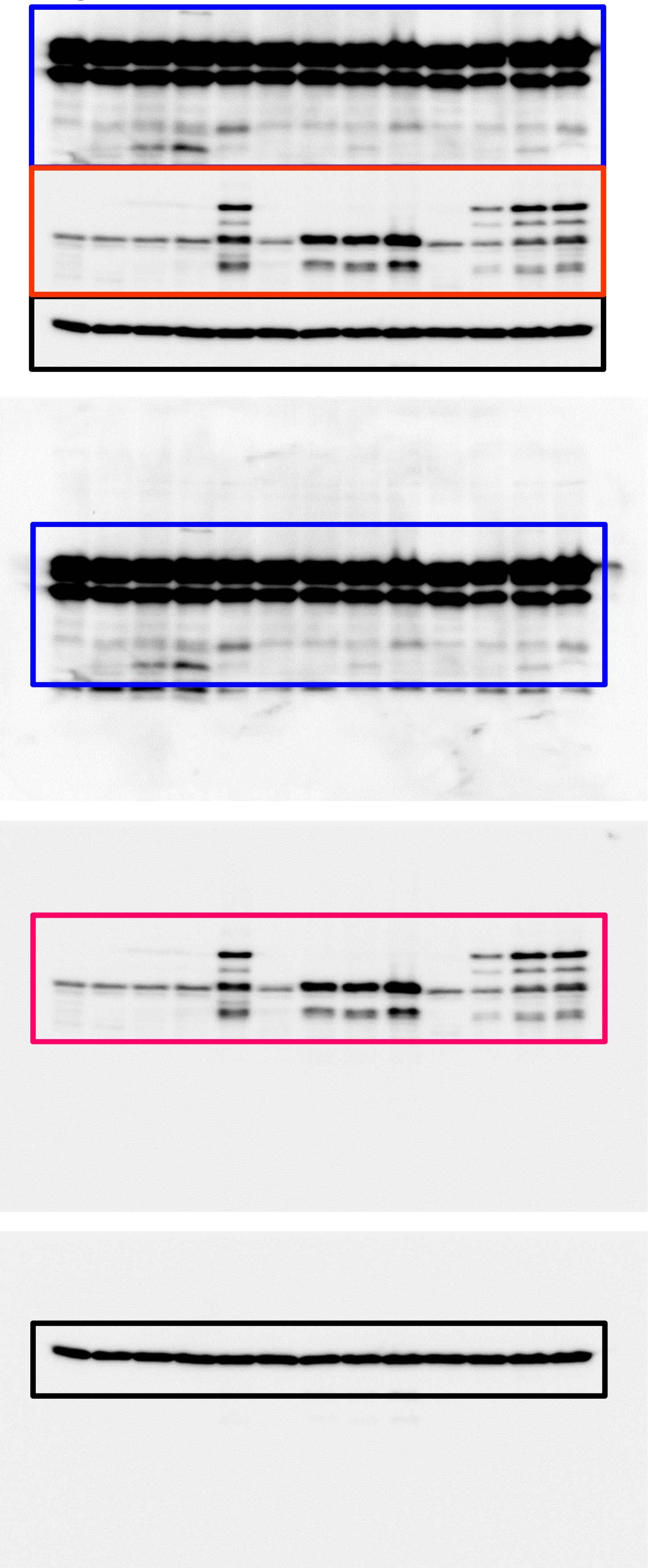

Fig. S2A – left, top

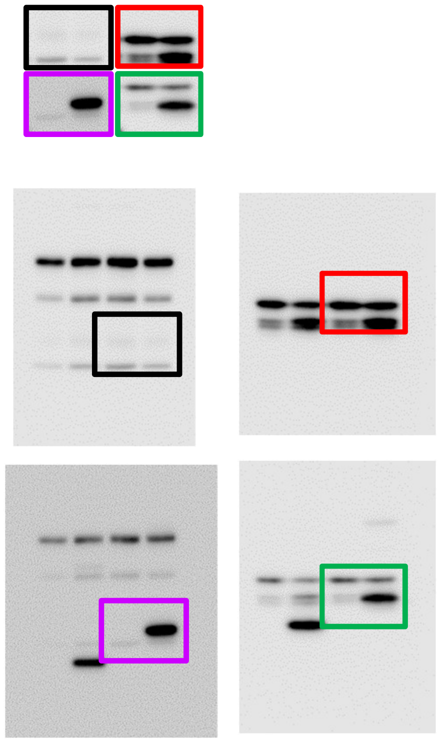

Fig. S2A – right, top

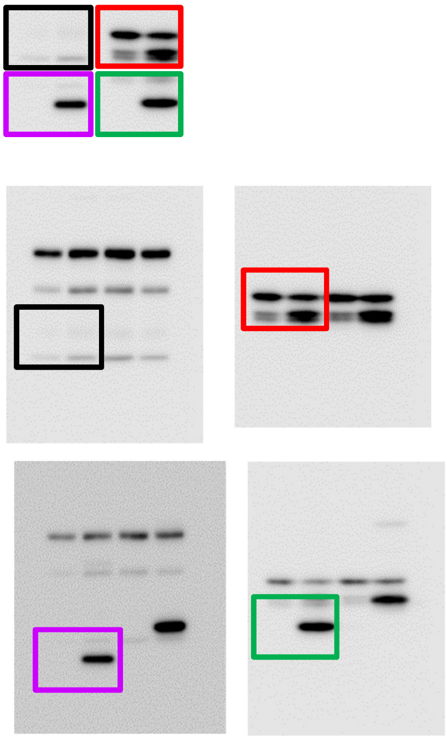

Fig. S2A – left, bottom

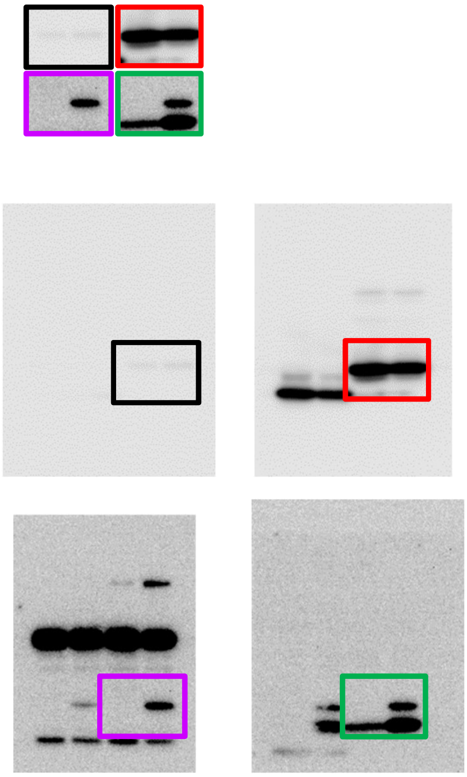

Fig. S2A – right, bottom

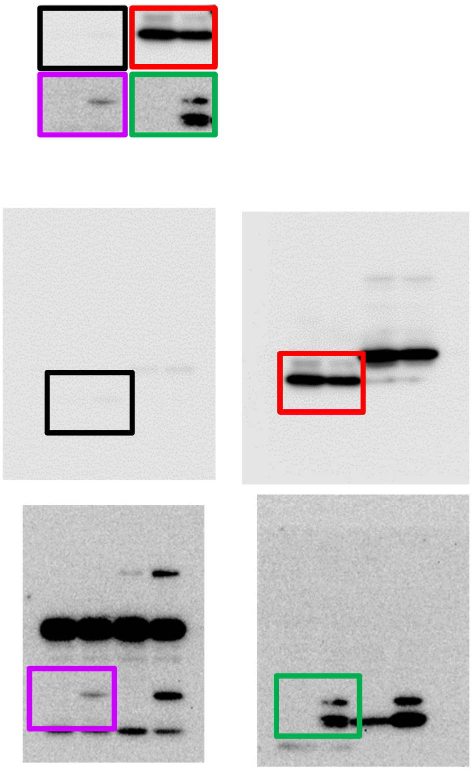

Fig. S2B – left, top

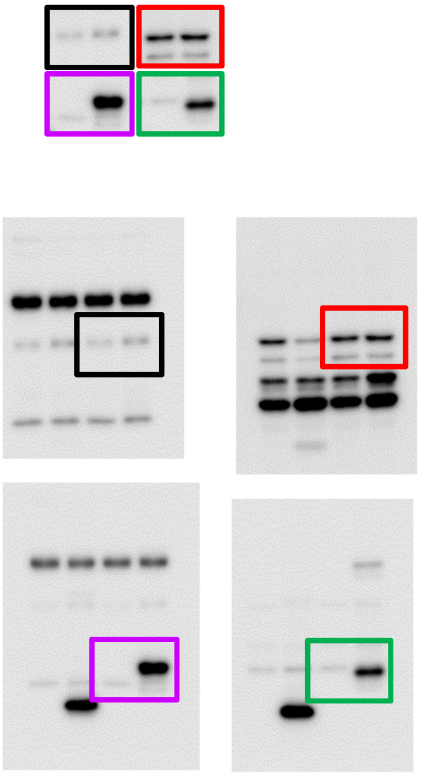

Fig. S2B – right, top

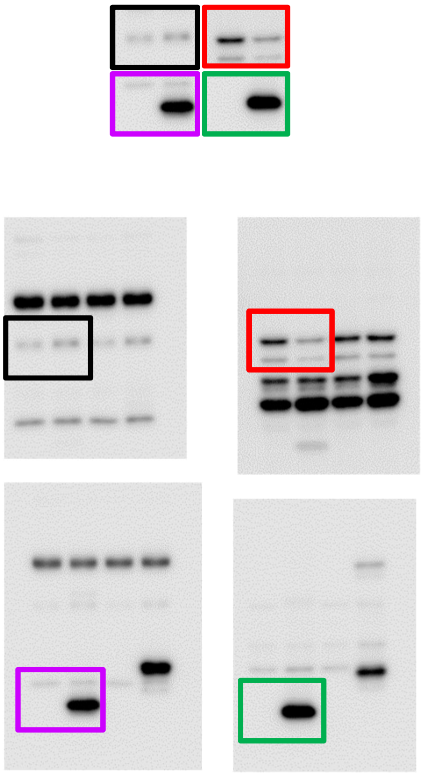

Fig. S2B – left, bottom

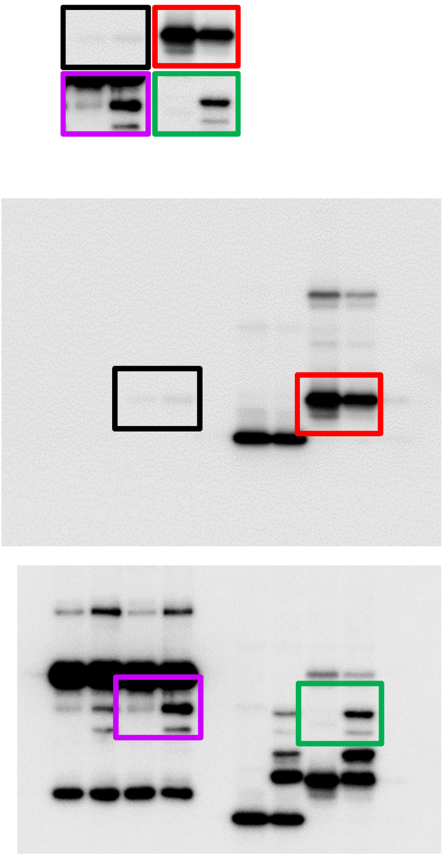

Fig. S2B – right, bottom

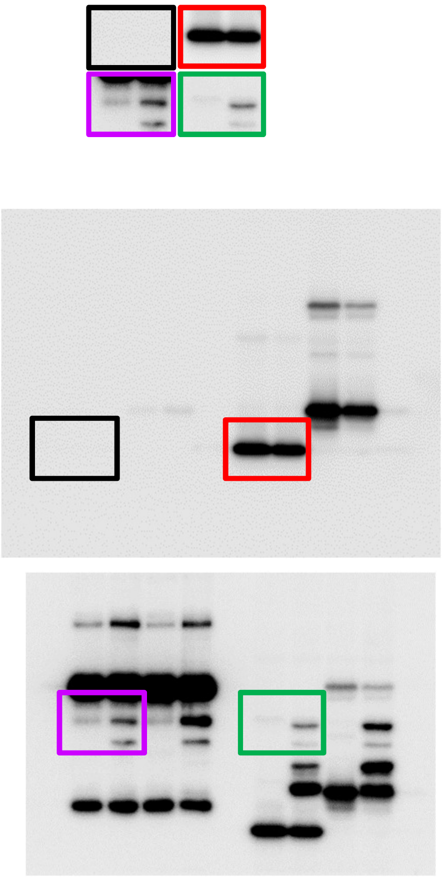

Fig. S3A

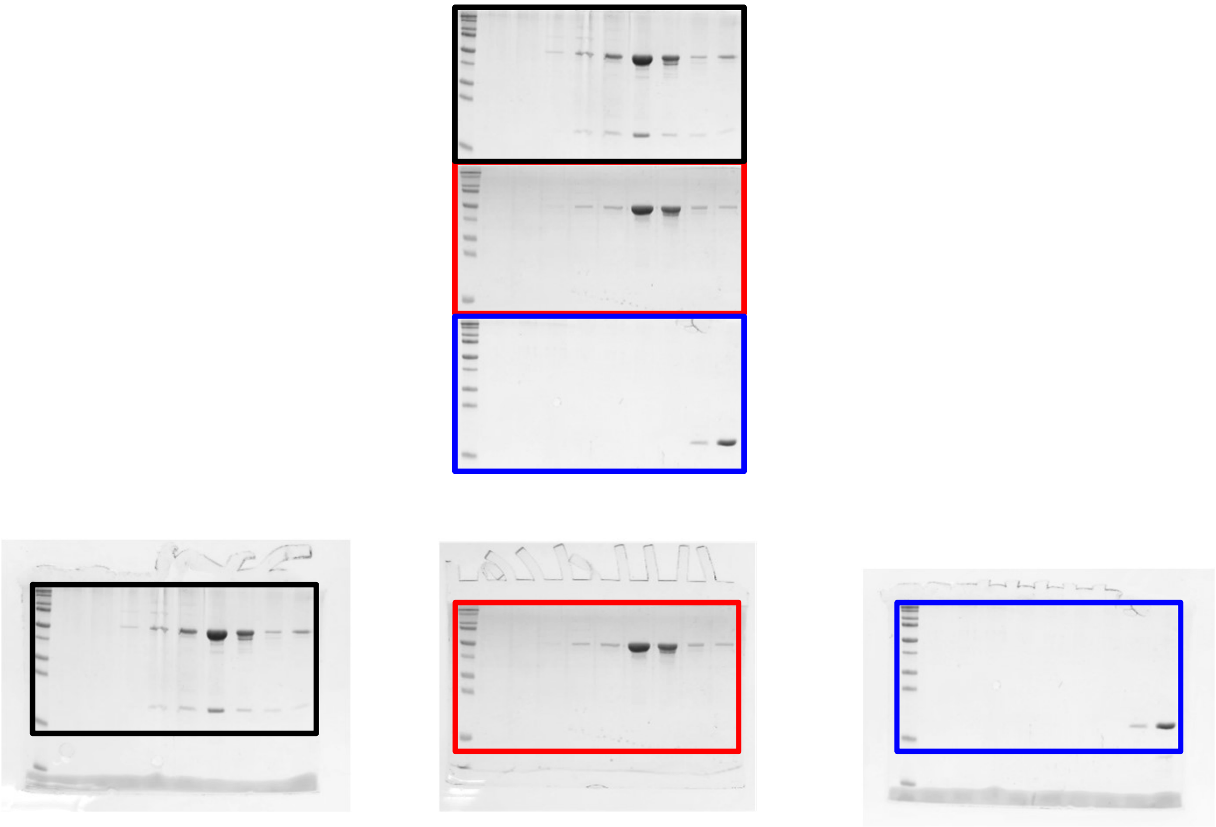

Fig. S6A – left

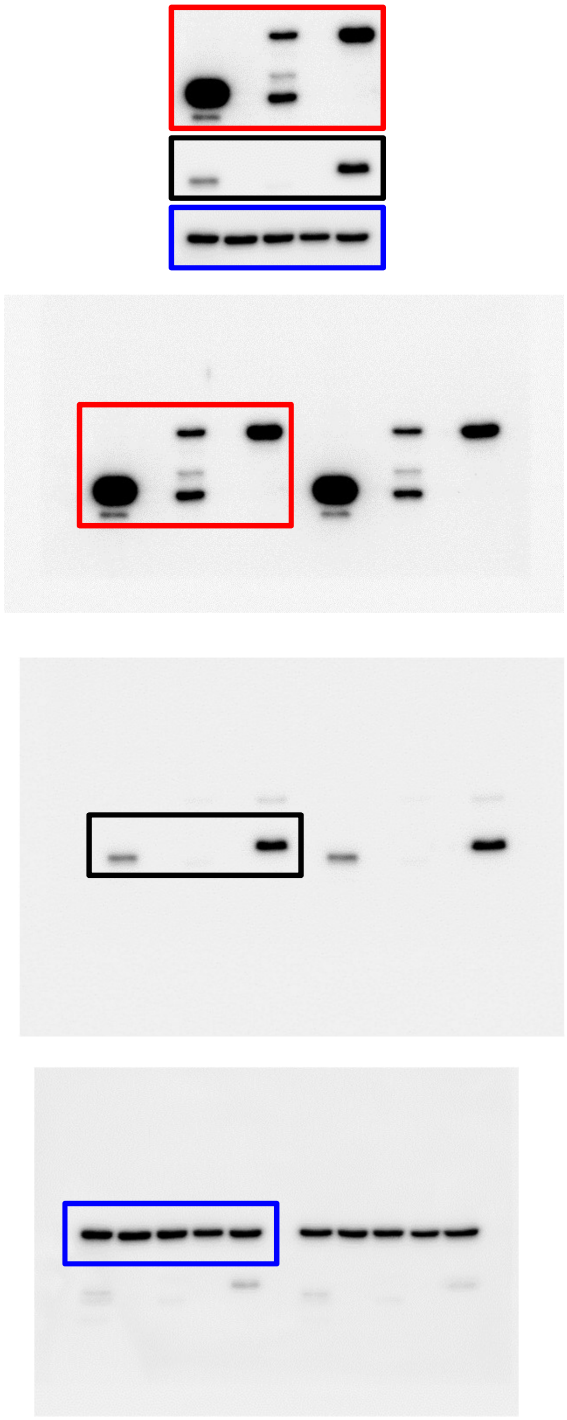

Fig. S6A – middle

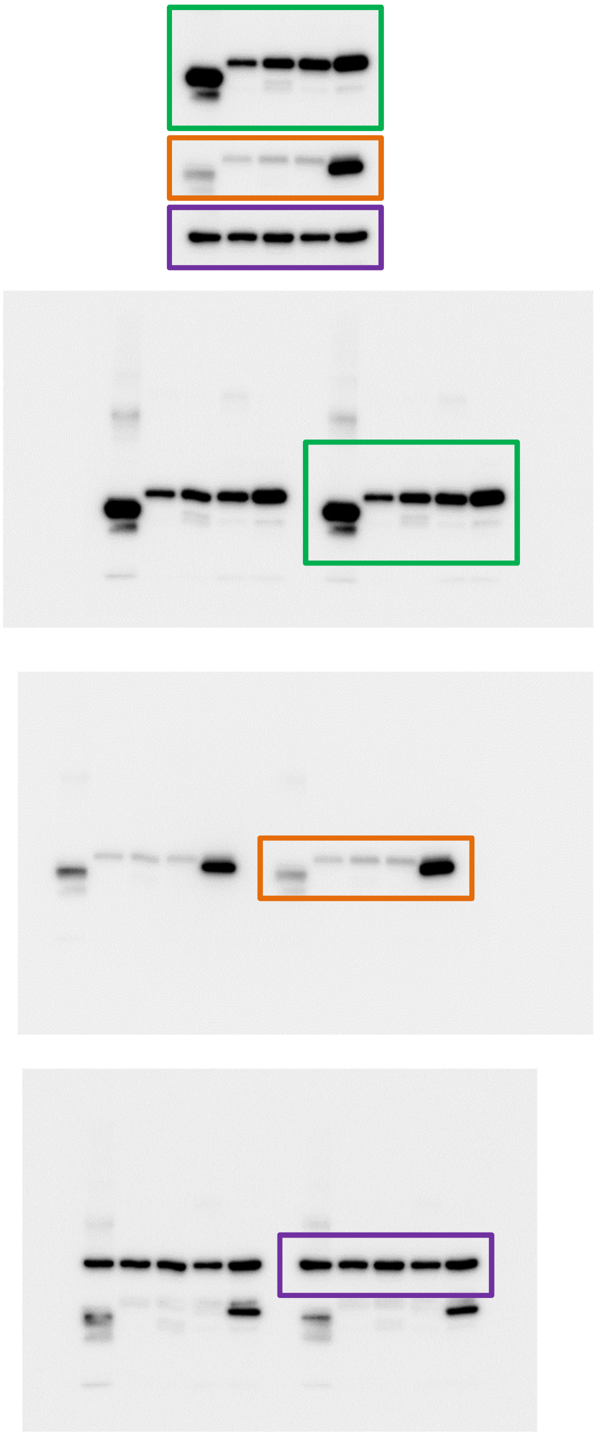

Fig. S6A – right

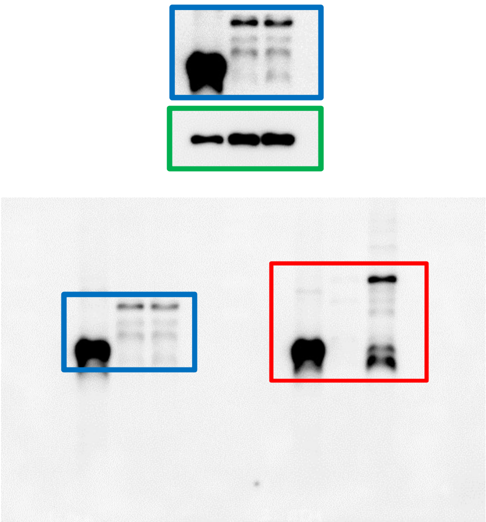

Fig. S6B

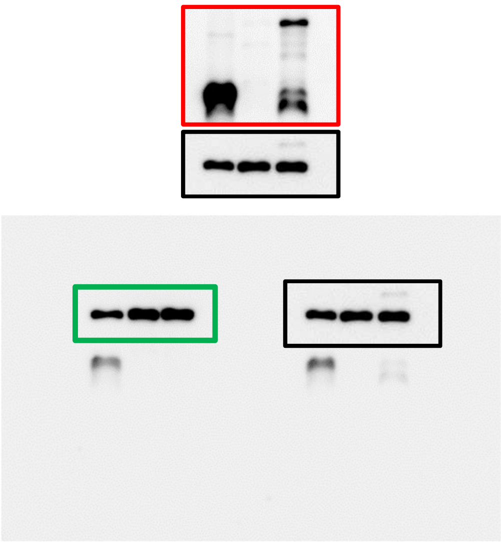

Fig. S6C – left

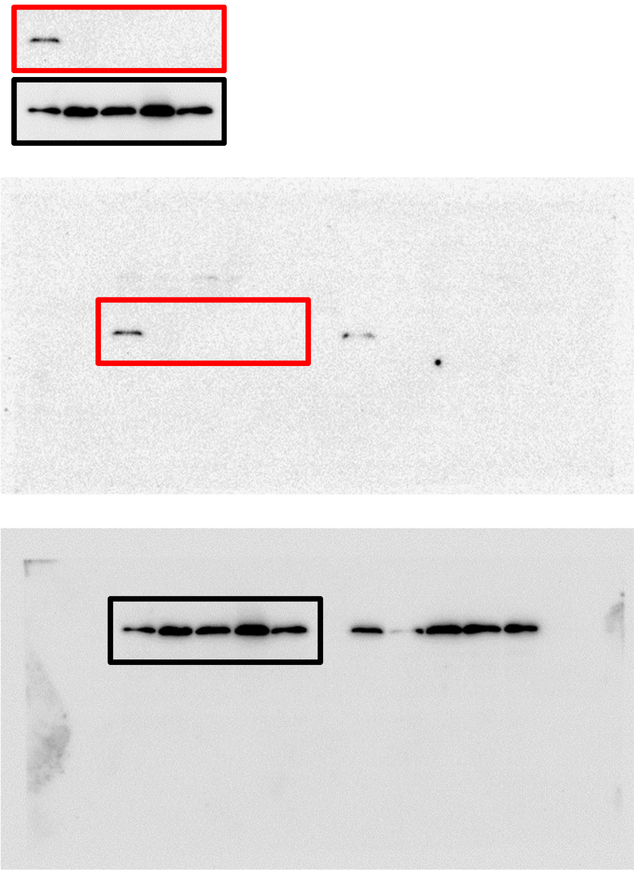

Fig. S6C – right

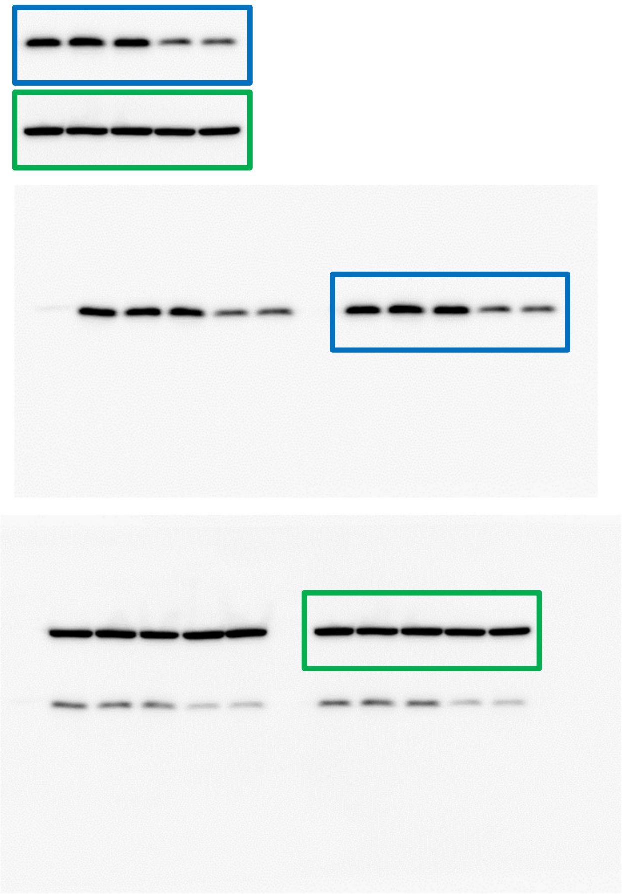

Fig. S6D

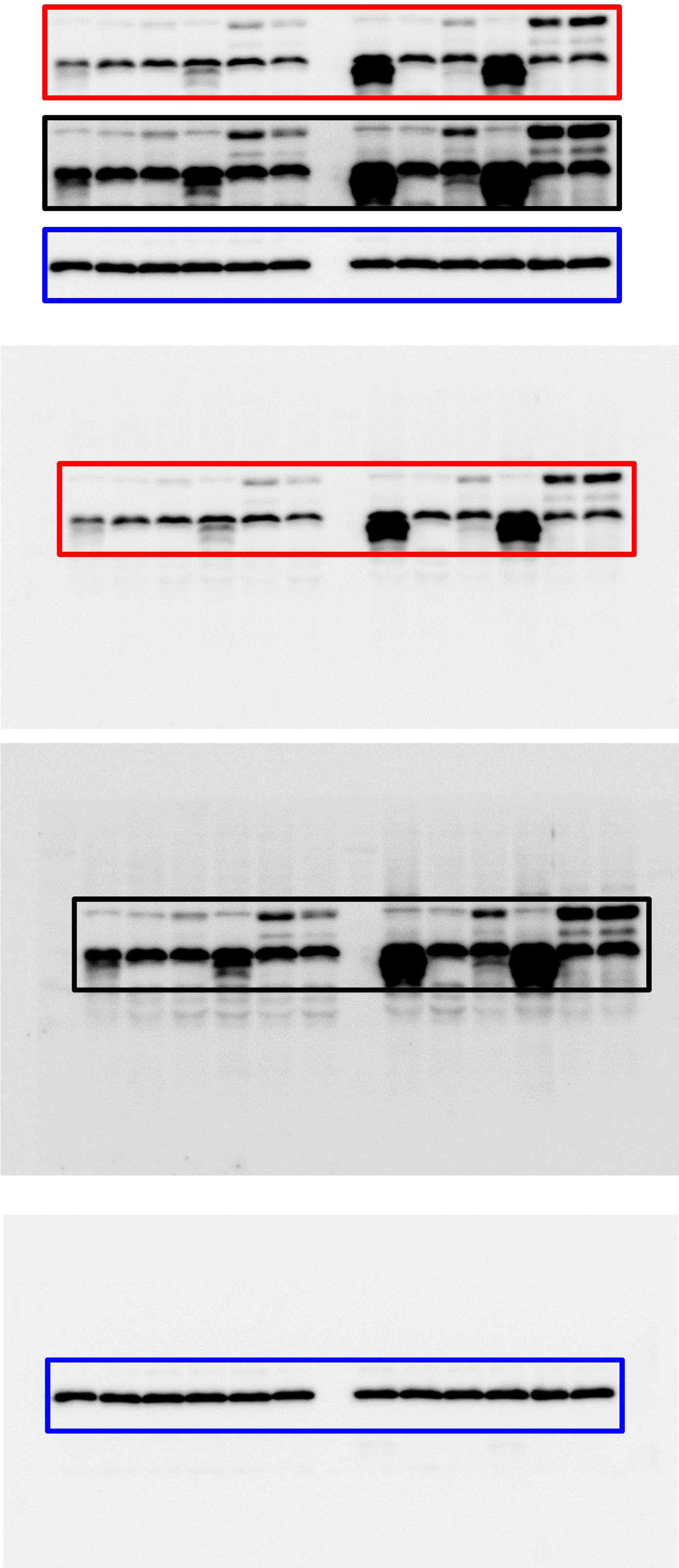

Fig. S7B

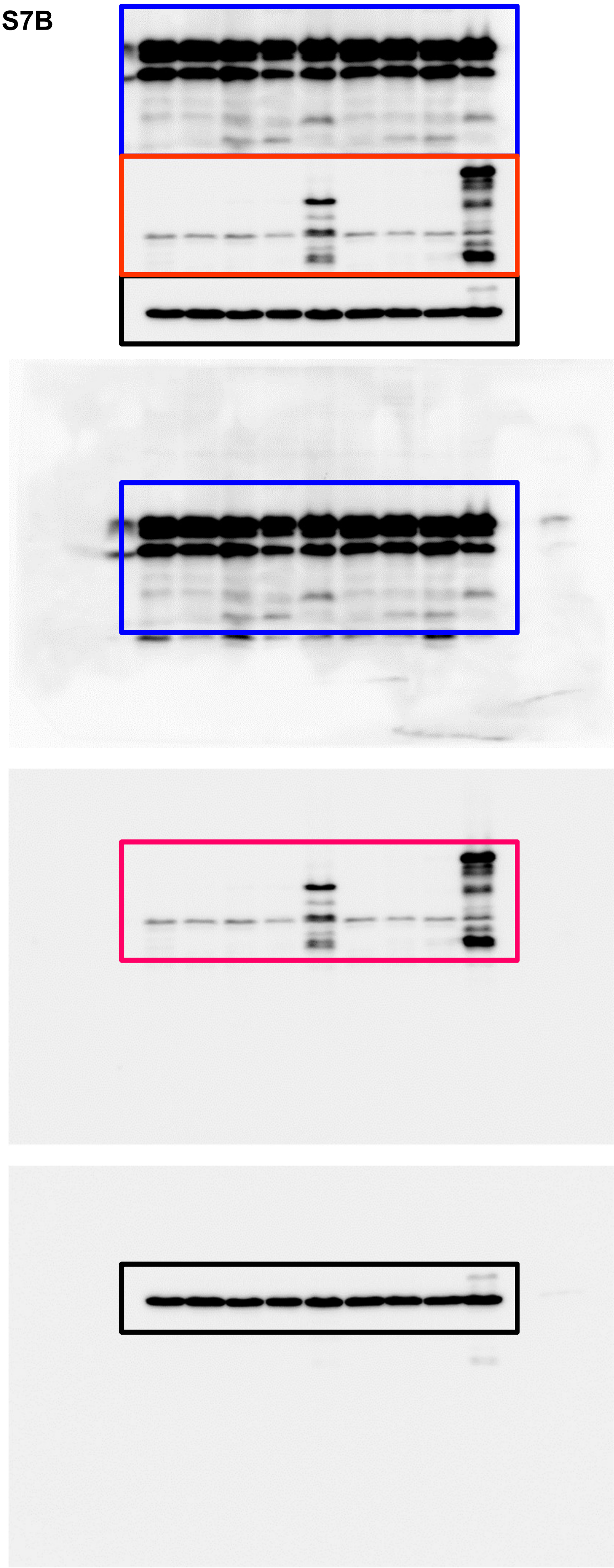

Supplement: S1 Raw Images — (PDF) [file pbio.3002156.s012.pdf]
