# Supplementary material for: Structural basis for proapoptotic activation of Bak by the noncanonical BH3-only protein Pxt1
Source: PLoS Biol. 2023 Jun 14;21(6):e3002156. doi: 10.1371/journal.pbio.3002156 (PMC10298792; doi:10.1371/journal.pbio.3002156)
Supplement: S1 Table — (DOCX) [file pbio.3002156.s013.docx]

**S1 Table. Data collection and structure refinement statistics**

| **Data Collection** | **PDB code 8GSV** |
| --- | --- |
| Space group | *P*1 |
| Unit cell dimensions |  |
| a, b, c (Å) | 70.00, 108.37, 108.42 |
| α, β, γ (^o^) | 120, 90, 90 |
| Resolution (Å) | 50.0−2.2 (2.24−2.20)^a^ |
| *R*_sym_^b^ (%) | 6.9 (28.8) |
| *I*/σ(*I*) | 14.4 (4.3) |
| Completeness (%) | 97.4 (96.9) |
| Redundancy | 3.6 |
| **Refinement** |  |
| Resolution (Å) | 50.0−2.2 |
| Number of reflections | 130,707 |
| *R*_work_^c^/*R*_free_ (%) | 21.1/26.6 |
| Number of atoms |  |
| Bak  Pxt1 | 14908  2144 |
| Water | 206 |
| Root-mean-square deviation |  |
| Bond lengths (Å) | 0.011 |
| Bond angles (^o^) | 1.097 |
| Ramachandran plot (%) |  |
| Most favored region | 94.0 |
| Additionally allowed region | 6.0 |
| Average B-values (Å^2^) |  |
| Bak  Pxt1 | 43.9  26.7 |
| Water | 24.5 |

^a^The numbers in parentheses are statistics from the shell with the highest resolution.

^b^*R*_sym_ = Σ |*I*_obs_ - *I*_avg_| / *I*_obs_, where *I*_obs_ is the observed intensity of individual reflection and *I*_avg_ is the average across symmetry equivalents.

^c^*R*_work_ = Σ ||*F*_o_| - |*F*_c_|| / Σ |*F*_o_|, where |*F*_o_| and |*F*_c_| are the observed and calculated structure factor amplitudes, respectively.
